# Spatiotemporal sphingosine-1-phosphate receptor 3 expression within the cerebral vasculature after ischemic stroke

**DOI:** 10.1016/j.isci.2024.110031

**Published:** 2024-05-20

**Authors:** Hana Matuskova, Lisa T. Porschen, Frank Matthes, Arne G. Lindgren, Gabor C. Petzold, Anja Meissner

**Affiliations:** 1 Department of Experimental Medical Sciences, Lund University, 221 84 Lund, Sweden; 2 Wallenberg Centre for Molecular Medicine, Lund University, 221 84 Lund, Sweden; 3 Division of Vascular Neurology, University Hospital Bonn, 53127 Bonn, Germany; 4 German Center for Neurodegenerative Diseases (DZNE), 53127 Bonn, Germany; 5 Department of Physiology, Institute of Theoretical Medicine, Medical Faculty, University of Augsburg, Augsburg, Germany; 6 Department of Clinical Sciences Lund, Neurology, Lund University, Lund, Sweden; 7 Department of Neurology, Rehabilitation Medicine, Memory Disorders and Geriatrics, Skåne University Hospital, Lund, Sweden

**Keywords:** Biological sciences, Molecular neuroscience, Natural sciences, Neuroscience, Pathophysiology, Physiology

## Abstract

Sphingosine-1-phosphate receptors (S1PRs) are promising therapeutic targets in cardiovascular disease, including ischemic stroke. However, important spatiotemporal information for alterations of S1PR expression is lacking. Here, we investigated the role of S1PR3 in ischemic stroke in rodent models and patient samples. We show that S1PR3 is acutely upregulated in perilesional reactive astrocytes after stroke, and that stroke volume and behavioral deficits are improved in mice lacking S1PR3. Further, we find that administration of an S1PR3 antagonist at 4-h post-stroke, but not at later timepoints, improves stroke outcome. Lastly, we observed higher plasma S1PR3 concentrations in experimental stroke and in patients with ischemic stroke. Together, our results establish S1PR3 as a potential drug target and biomarker in ischemic stroke.

## Highlights

• S1PR3 is upregulated in perilesional reactive astrocytes acutely after stroke
• Stroke volume and behavioral deficits are improved in mice lacking S1PR3
• Therapeutically antagonizing S1PR3 improves stroke outcome
• Circulating S1PR3 levels increase after experimental and human ischemic stroke

## Graphical abstract

**Figure:**
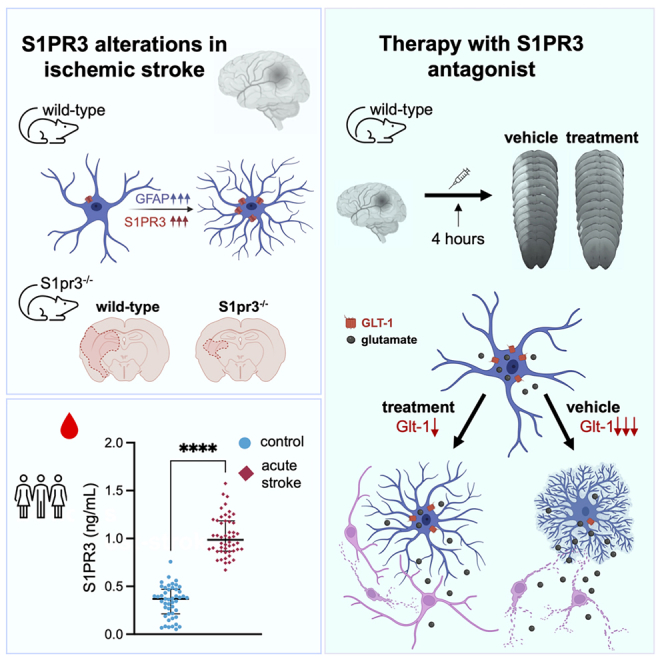

## Introduction

Alterations in the signaling of the bioactive lipid sphingosine-1-phosphate (S1P) have been associated with various cardiovascular diseases, including stroke.^1^^,^^2^ By binding to five G protein-coupled S1P receptors (S1PR1–5), S1P is involved in an array of physiological functions^3^^,^^4^^,^^5^ but also mediates several pathophysiological responses.^6^^,^^7^^,^^8^^,^^9^ S1P signaling has emerged as a new biomarker and promising therapeutic target for cardiovascular pathologies, including ischemic brain damage.^10^^,^^11^^,^^12^^,^^13^ The non-selective S1PR modulator fingolimod, approved for relapsing-remitting multiple sclerosis, is currently in clinical stroke trials (ClinicalTrials.gov Identifier: NCT04629872, NCT04718064, NCT02002390, and NCT04088630), despite inconsistent results in preclinical studies of experimental stroke.^14^^,^^15^^,^^16^^,^^17^ Fingolimod exerts its effects on four of the five S1PRs that are ubiquitously expressed throughout all bodily tissues with often organ- and cell-specific signaling,^18^^,^^19^ which may explain these discrepancies. Hence, there is a translational and clinical need to better understand S1P signaling after stroke before S1P-specific targets can be applied to stroke therapy.

To better understand relevant S1PR signaling post-stroke, we specifically investigated S1PR3 responses during ischemic brain damage in experimental mouse models and in patients with ischemic stroke. Especially in the context of stroke, S1PR3 is less studied compared to S1PR1 and S1PR2 although previous studies showed that S1PR3 rapidly increases post-ischemia^20^^,^^21^ and is majorly involved in maintenance of vascular integrity.^3^ Our goal was to explore cell-specific S1PR3 alterations post-stroke with the focus on blood-brain barrier (BBB) constituents.

## Results

### Experimental stroke is associated with S1PR3 upregulation in astrocytes

**Figure 1:**
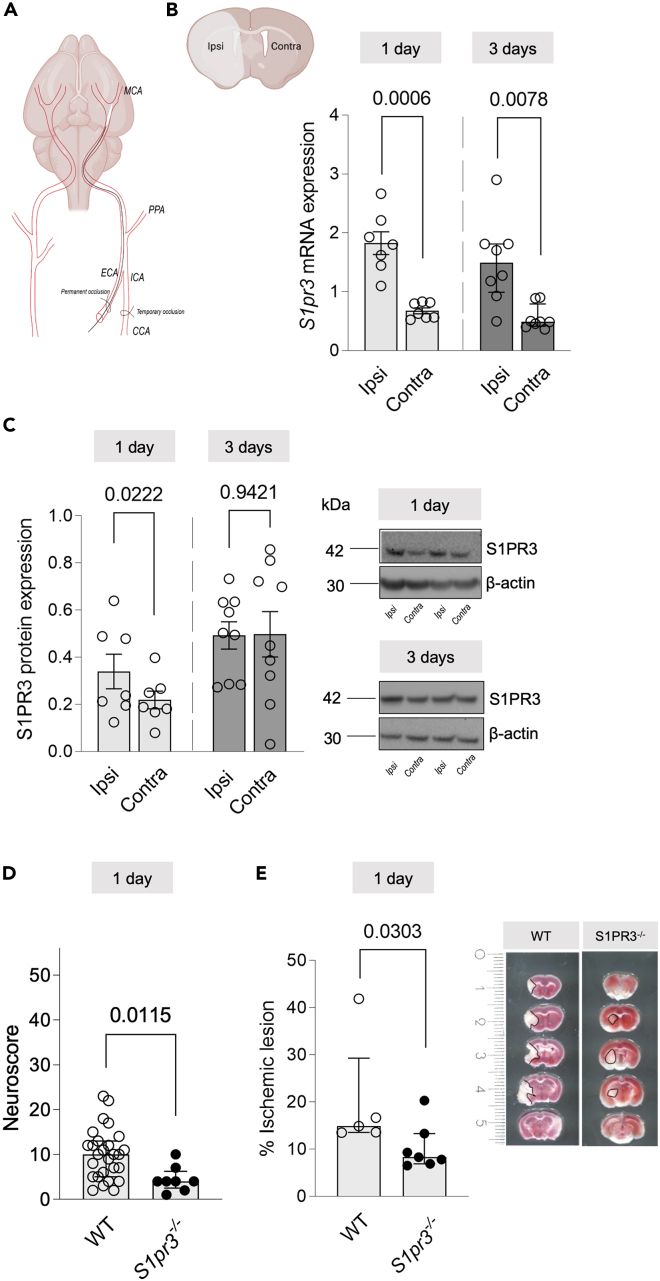
Ipsilateral *S1pr3* expression is augmented post-stroke (A) Schematic illustration of transient middle cerebral artery occlusion (tMCAo) to induce ischemic stroke in mice. A filament was inserted into the left MCA to block blood flow. After 60 min reperfusion was induced by removing the filament. In comparison to contralateral (contra) hemispheres, tMCAo increases ipsilaterally (ipsi) (B) mRNA expression of sphingosine-1-phosphate receptor 3 *(S1pr3*) 1-day (*n* = 7) and 3-days (*n* = 8) post-stroke, and (C) S1PR3 protein expression 1-day (*n* = 7) but not 3-days (*n* = 9) post-stroke. Related to Figure S1. Representative images show *S1pr3* expression in whole tissue extracts from ipsi- and contralateral hemispheres. *S1pr3* knockout mice (*S1pr3*^*−/−*^) presented with (D) lower neuroscore (WT: *n* = 27, *S1pr3*^*−/−*^*: n* = 8*)* and (E) smaller infarct lesions compared to WT mice (WT: *n* = 5, *S1pr3*^*−/−*^*: n* = 7*)* 1-day post-stroke. Related to Figure S2. (B) (3-days), (D), and (E) are presented as median ± interquartile range and were compared with Wilcoxon (B) or Mann-Whitney (D and E). (B) (1-day) and (C) are presented as mean ± SEM and are compared with a Student’s t test. Exact *p* values are given for all comparisons. Scale bar is in cm in (E). Schematic illustrations created with BioRender.

**Figure 2:**
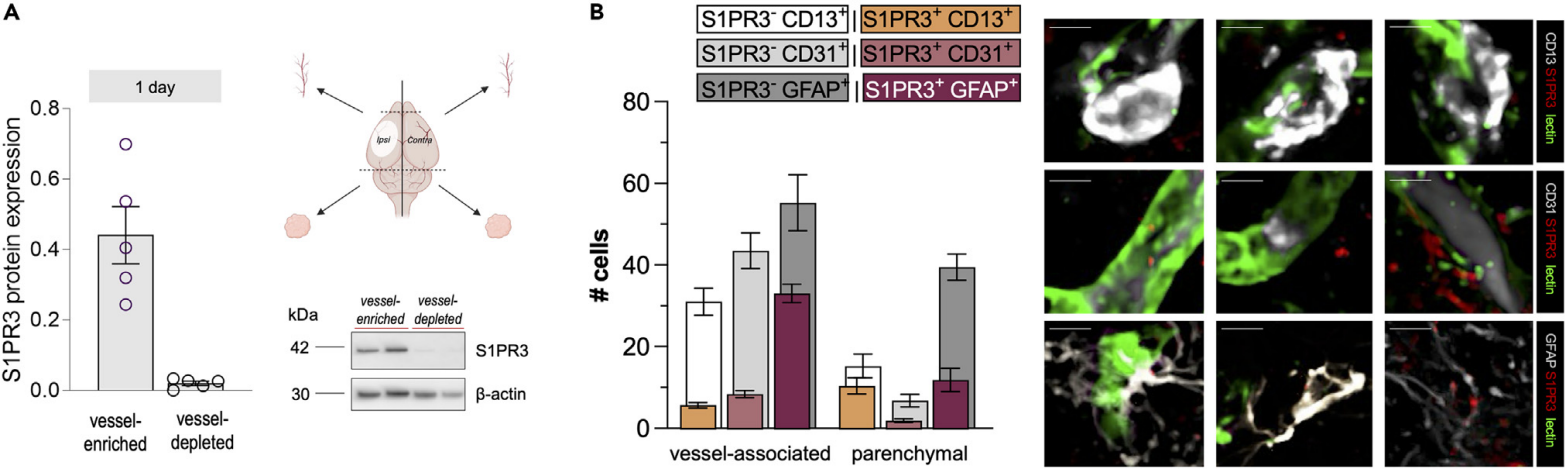
S1PR3 is mainly expressed by vessel-associated astrocytes (A) The majority of S1PR3 associates with the vessel-rich fraction at 1-day post-tMCAo (*n* = 5; quantification shown in the graph and representative image). Schematic illustration of a brain tissue fractionation approach to determine vascular localization of S1PR3 protein expression in the brain. (B) Quantification of vessel-associated and non-vessel associated S1PR3 positivity of CD13^+^, CD31^+^, and GFAP^+^ cells showing the majority of S1PR3 co-occurring with vessel-associated GFAP positivity. Representative images showing S1PR3 positivity in CD13^+^, CD31^+^ and GFAP^+^ cells. Data presented as mean ± SEM. Bar over micrographs is 50 μm in (B). Schematic illustrations created with BioRender.

**Figure 3:**
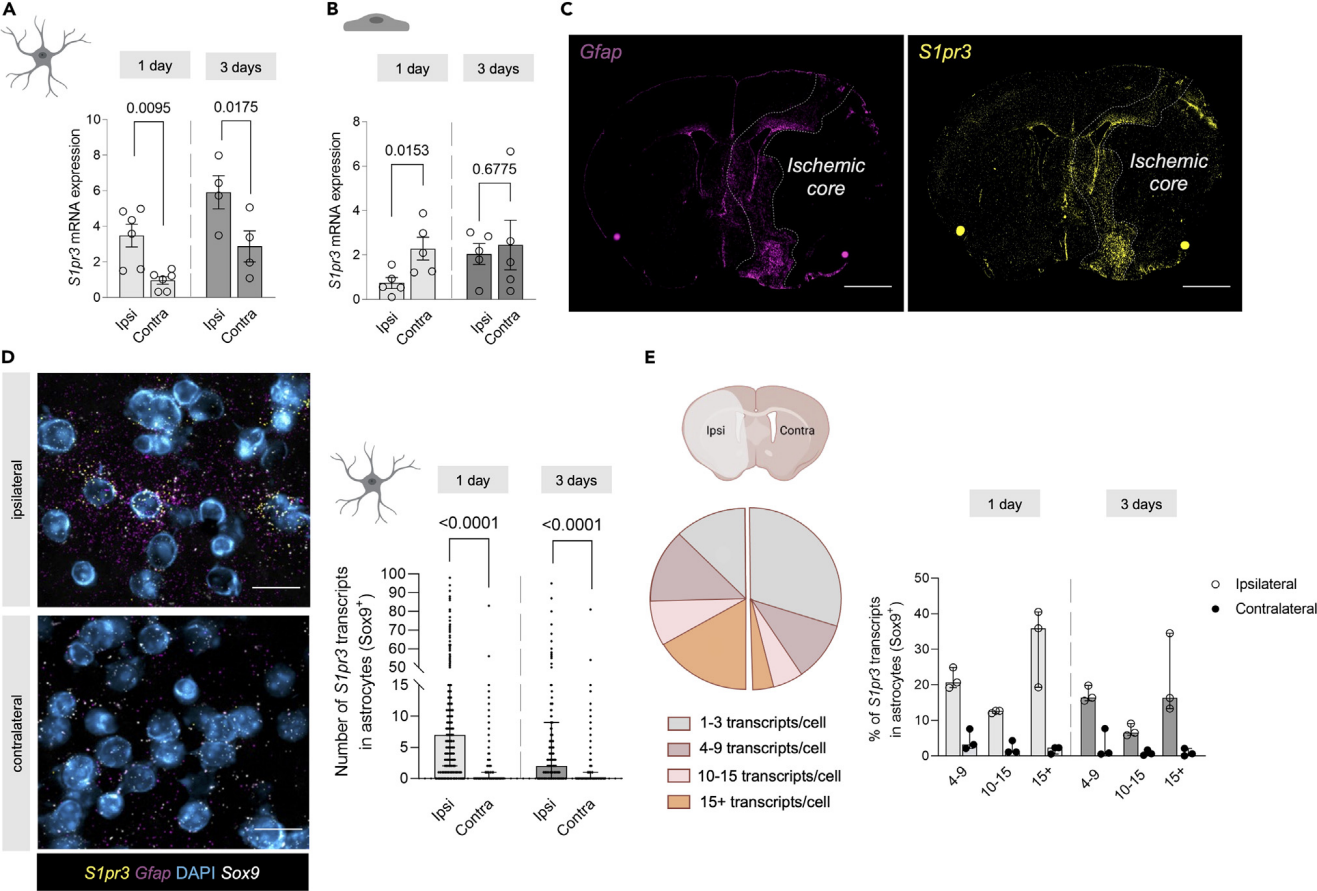
Astrocytes are main contributors to higher cerebrovascular *S1pr3* expression post-stroke (A) Transient middle cerebral artery occlusion (tMCAo) in *Cnx43*^*Cre-ER(T)*^/RiboTag mice leads to increased astrocyte-specific sphingosine-1-phosphate receptor 3 (*S1pr3*) expression in the ipsilateral (ipsi) hemisphere 1-day and 3-days post-stroke (*n* = 6 and *n* = 4, respectively). Related to Figure S3. (B) tMCAo in *Cdh5*^*Cre-ER(T)*^/RiboTag mice causes reduction of endothelial cell-specific *S1pr3* expression ipsilaterally 1-day post-stroke (*n* = 5) but not 3-days post-stroke (*n* = 5). (C) Representative overview of brain slices illustrating mRNA transcript locations of *Gfap* (purple) and *S1pr3* (yellow) 3-days post-stroke and revealing colocalization within the glial scar. (D) Representative images illustrating RNA transcripts of *Sox9* (white), *Gfap* (purple), and *S1pr3* (yellow) around DAPI-stained nuclei (blue) in the brain 3-days post-stroke. Quantification shows increased ipsilateral (ipsi) transcript numbers of *S1pr3* per *Sox9*^+^ cell 1-day (*n* = 3) and 3-days post-stroke (*n* = 3) compared to the contralateral (contra) hemisphere. (E) Categorization of *Sox9*^+^ cells according to *S1pr3* transcript numbers shows higher numbers of *S1pr3*^+^ cells in ipsilateral vs. contralateral hemispheres at 1-day or 3-days post-stroke (*n* = 3). Related to Figures S4 and S5. (D) and (E) are presented as median ± interquartile range and (D) is compared with a Wilcoxon test. (A) and (B) are presented as mean ± SEM and are compared with a Student’s t test. Exact *p* values are given for all comparisons. Bar over micrographs is 1 mm in (C) and 50 μm in (D). Schematic illustrations created with BioRender.

To investigate temporal changes of S1PR3 expression post-stroke, we used a murine model of transient middle cerebral artery occlusion (tMCAo; Figure 1A), mimicking a clinical scenario with induced reperfusion. *S1pr3* mRNA expression was significantly upregulated in the ipsilateral (lesioned) hemisphere compared to the contralateral (control) hemisphere at 1-day and 3-days post-tMCAo (Figure 1B) as well as compared to sham-operated mice (Figures S1A and S1B). S1PR3 protein expression was elevated only at 1-day post-tMCAo (Figure 1C). As S1PR3 can mediate both beneficial and detrimental effects in non-cerebral ischemia/reperfusion injuries,^22^^,^^23^^,^^24^^,^^25^ we subjected *S1pr3* knockout mice (*S1pr3*^−/−^; Figure S2A) to tMCAo. Neurological function evaluated by using extended neuroscore testing^26^ revealed a significantly better neurological outcome post-stroke in *S1pr3*^−/−^ mice compared to wild-type (WT) mice (Figure 1D). This was further associated with smaller infarct lesion sizes assessed by TTC (2,3,5-triphenyltetrazolium chloride) staining (Figure 1E), suggesting beneficial effects of dampened S1PR3 expression after stroke. To identify cell-specific contributions to the S1PR3 elevation occurring acutely post-stroke, we performed brain tissue separation into vessel-rich and vessel-depleted fractions from contra- and ipsilateral hemispheres at 1-day after tMCAo.^27^ Evaluation of S1PR3 protein expression in both fractions revealed an exclusive association of S1PR3 with the vessel-rich fraction (Figure 2A), suggesting several cell types forming the BBB as contributors to elevated S1PR3 expression post-stroke. Indeed, S1PR3 positivity was observed for CD13^+^ pericytes, CD31^+^ endothelial cells and GFAP^+^ astrocytes with most prominent presence in vessel-associated astrocytes (Figure 2B). As astrocytes and endothelial cells highly express S1PR3 in the healthy mouse brain,^28^ we next assessed the astroglial and endothelial translatome after stroke using RiboTag mouse lines with tamoxifen-induced expression of a hemagglutinin tag on the ribosomal protein subunit under control of astrocyte-specific connexin 43 (*Cnx43*^Cre−ER(T)^/RiboTag) or endothelial-specific cadherin 5 (*Cdh5*^Cre−ER(T)^/RiboTag) promotors.^29^ Astrocyte-specific *S1pr3* mRNA levels, as confirmed by enrichment of the expression of astrocytic markers such as *Aldh1l1*, *Gfap*, and *Slc1a2* (Figures S3A–S3D), significantly increased in the ipsilateral compared to the contralateral hemisphere (Figure 3A) and to sham surgeries (Figures S4A and S4B). To illustrate S1PR3 predominance in astrocytes under these conditions, we verified higher astrocyte-specific ipsi-contra ratios of *S1pr3* expression when comparing to whole tissue extracts (5.1-fold vs. 2.6-fold at 1-day and 2.6-fold vs. 1.9-fold at 3-days post-stroke; Figure S4C). In contrast to astrocytes, endothelial-specific *S1pr3* expression was decreased in the ipsilateral hemisphere at 1-day after tMCAo; and no differences between the hemispheres were detected at 3-days post-tMCAo (Figure 3B). Accordingly, *S1pr3* ipsi-contra ratios were lower in endothelial cells compared to whole brain extracts (0.3-fold vs. 3.9-fold at 1-day and 1.3-fold vs. 2.5-fold at 3-days post-stroke; Figure S4D). A direct comparison of astrocyte and endothelial cell *S1pr3* expression verified an ipsilateral upregulation in astrocytes but not endothelial cells at 3-days post-tMCAo (Figure S4E). We next performed *in situ* hybridization to qualitatively confirm these findings and spatially localize cell-specific *S1pr3* expression. Whole brain images visualized with probes for glial fibrillary acidic protein (*Gfap*), a marker of reactive astrocytes,^30^ and *S1pr3* revealed a prominent colocalization within the glial peri-infarct scar (Figure 3C). As *Gfap* expression rapidly increases unilaterally in the ischemic hemisphere (Figures S4F and S4G), we used the pan-astrocytic marker SRY-box transcription factor 9 (*Sox9*)^31^ for quantitative comparisons between the ipsi- and contralateral hemispheres (*Sox9* ipsi:contra ratios in tMCAo vs. sham controls: 1.007 ± 0.031 vs. 0.962 ± 0.093). This analysis showed that *Sox9*-positive astrocytes carried an increased number of *S1pr3* transcripts in the ipsilateral hemisphere at 1-day and 3-days after tMCAo (Figure 3D). Categorization of *S1pr3* expressing astrocytes illustrated an abundance of astrocytes with 4–9, 10–15, and >15 *S1pr3* transcripts per cell in the ischemic hemisphere at both timepoints after tMCAo, while the number of astrocytes with less than four *S1pr3* transcripts per cell was lower compared to the non-lesioned hemisphere (Figure 3E). In contrast to astroglial *S1pr3* expression, *CD31*-positive endothelial cells presented with an increased number of *S1pr3* transcripts in the ipsilateral hemisphere only 3-days after tMCAo (Figure S5A). Categorization of *S1pr3* expressing endothelial cells, however, showed no differences in *S1pr3* transcript abundance per cell between ischemic and control hemispheres at both timepoints after tMCAo (Figure S5B). In summary, upregulation of S1PR3 expression in a model of tMCAo is adversely related to neurological outcome and infarct lesion. Further cell-specific investigation localized increased *S1pr3* expression in the glial scar and moreover, confirmed acute astrocyte-specific augmentation of *S1pr3* expression after ischemic stroke in mice.

### Therapeutically antagonizing S1PR3 improves stroke outcome

**Figure 4:**
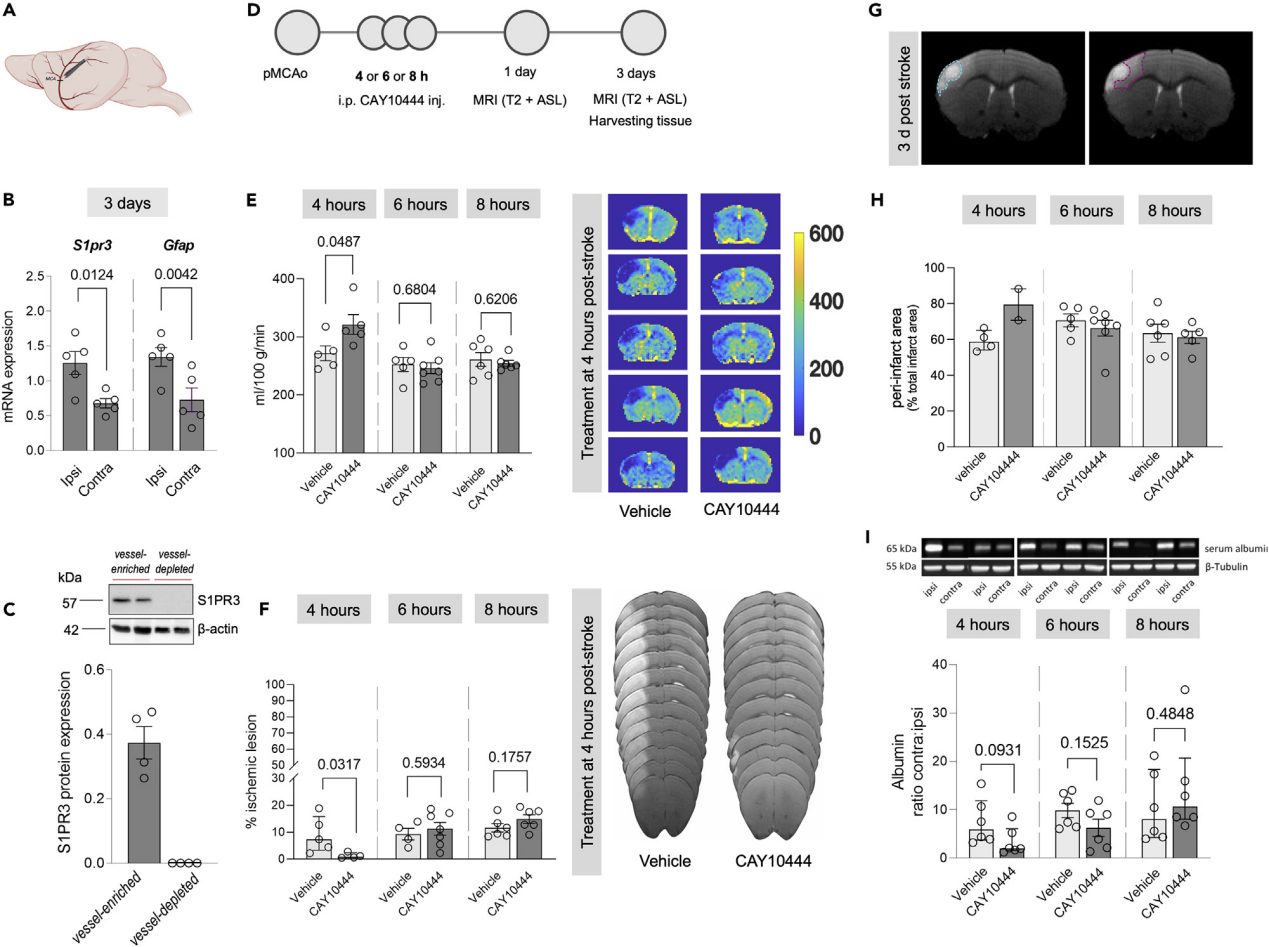
Therapeutically antagonizing S1PR3 improves stroke outcome dependent on timepoint of treatment initiation (A) Schematic illustration of permanent middle cerebral artery occlusion (pMCAo) to induce ischemic stroke without reperfusion in mice. (B) pMCAo increases ipsilateral (ipsi) mRNA expression of sphingosine-1-phosphate receptor 3 (*S1pr3*) and glial fibrillary acidic protein (*Gfap*) 3-days (*n* = 5) post-stroke compared to the respective contralateral (contra) hemispheres. (C) Brain tissue fractionation reveals vascular localization of S1PR3 expression in the brain (*n* = 4). (D) Illustration of experimental timeline. (E) Administration of the S1PR3 antagonist CAY10444 improves total cerebral blood flow 3-days post-stroke only when given at 4-h post-stroke (4-h: *n* = 5; 6-h: vehicle – *n* = 5, CAY10444 – *n* = 7; 8-h: *n* = 6). Representative arterial spin labeling (ASL) map images for treatments initiated at 4-h post-stroke. (F) Administration of the S1PR3 antagonist CAY10444 reduces overall infarct lesion size 3-days post-stroke only when given at 4-h post-stroke. Analysis of the infarct lesion of CAY10444-treated group was performed only with four mice per group as the value from one mouse was excluded using ROUT method of removing outliers (4-h: *n* = 5; 6-h: vehicle – *n* = 4, CAY10444 – *n* = 7; 8-h: *n* = 6). Representative T2 map images of infarct lesions for treatment initiated at 4 h post-stroke. (G) Representative brain magnetic resonance images showing infarct lesion (white region) with areas of different water content highlighted with blue (ischemic core) and violet (peri-infarct) dashed lines. (H) Quantified peri-infarct proportions within the ischemic lesion in stroke mice after S1PR3 antagonist CAY10444 or vehicle treatment (4-h: vehicle - *n* = 4, CAY10444 – *n* = 2; 6-h: vehicle – *n* = 5, CAY10444 – *n* = 7; 8-h: vehicle - *n* = 6, CAY10444 – *n* = 5). Related to Figures S6–S9. (I) Effects of S1PR3 antagonist treatment on serum albumin accumulation in the vessel-depleted brain fraction 3-days post-stroke quantified as ipsi:contra ratios. Representative western blot images showing ipsi- and contralateral serum albumin after different treatment initiation timepoints. (B), (E), (F), (H), and (I) (6-h) are presented as mean ± SEM and were compared with a Student’s t test. (I) (4- and 8-h) is presented as median ± interquartile range and was compared with a Mann-Whitney test with calculated *p* values given per comparison. (C) and (H) are not subjected to statistical testing. Exact *p* values are given for all comparisons. Schematic illustrations created with BioRender.

A large proportion of ischemic stroke patients are not eligible for reperfusion treatment.^32^ Therefore, we also evaluated S1PR3 changes in a stroke model with permanent MCA occlusion (pMCAo; Figure 4A). Similar to the reperfusion model, *S1pr3* and *Gfap* gene expressions were upregulated at 1-day and 3-days post-pMCAo (Figures 4B and S6A). Likewise, S1PR3 protein expression was solely associated with the cerebral vessel-rich fraction (Figure 4C), suggesting involvement of S1PR3 signaling in ischemic brain damage independent of reperfusion. Specifically, therapeutic S1PR3 antagonism attenuated the increase of ipsilateral S1PR3 expression as evidenced by lower ipsi:contra ratios in the treated group compared to the vehicle group (Figure S7A). Additional analysis of the vessel-depleted fraction suggests the involvement of S1PR3 in stroke-associated ipsilateral GFAP upregulation in vessel-associated cells as GFAP ipsi:contra ratios were similar between S1PR3-antagonist and vehicle treated mice in vessel-depleted brain tissue, while the vessel-rich compartment revealed lower GFAP ipsi:contra ratios in the S1PR3-antagonist group compared to vehicle (Figure S7B). To further assess the therapeutic potential of S1PR3 antagonization, we subjected mice to single intraperitoneal (i.p.) injections of the S1PR3 antagonist CAY10444 (1 mg/kg) at different timepoints post-stroke (Figure 4D). A single CAY10444 injection given at 4-h after pMCAo resulted in a significant improvement of overall cerebral perfusion assessed by arterial spin labeling (ASL) using magnetic resonance (MR) imaging and reduced the percentage of infarct lesion at 3-days post-pMCAo (Figures 4E, 4F, and S6B–S6E). To explore the therapeutic time window, separate cohorts of mice were subjected to single injections at 6- or 8-h after stroke surgery. Later administration had no beneficial effect on overall tissue perfusion or total infarct volume (Figures 4E and 4F). Because most T2-weighted MR images presented with heterogeneous signal intensities suggestive of different degrees of tissue damage^33^ (Figure 4G), we determined the peri-infarct percentage of total infarct area. We defined the area with the highest T2 signal within the ischemic lesion as “core” and termed the area with less than 80% of the core T2 signal intensity as “peri-infarct area” (Figures S8A–S8C and S9). In mice treated with CAY10444 at 4-h post-stroke only two of five had a visible peri-infarct area. Here, the proportion of peri-infarct area was higher compared to vehicle treated mice (79.48 ± 8.760 vs. 59.27 ± 2.857; Figure 4H). Later administration yielded peri-infarct proportions that did not statistically differ from those of vehicle controls (Figure 4H). Since administration of tissue plasminogen activator later than 4.5-h post-stroke is associated with increased risk of hemorrhagic transformation,^34^ we investigated potential side effects of later CAY10444 administration. Following fractionation into vessel-rich and vessel-depleted tissue, we assessed the presence of serum albumin, indicative of BBB leakage and potential vessel disruption,^35^ in the vessel-depleted brain fractions of mice that received CAY10444 injection at 4-, 6-, or 8-h post-pMCAo. Albeit not reaching statistical significance, the lowest serum albumin levels in the vessel-depleted brain fractions were detected in mice that received CAY10444 injection at 4-h post-pMCAo (2.2-fold lower ipsi-contra level ratio compared to vehicle; Figure 4I) and at 6-h post-pMCAo (1.6-fold lower ipsi-contra level ratio compared to vehicle; Figure 4I). Mice receiving CAY10444 injection at 8-h post-pMCAo showed no differences in ipsilateral serum albumin levels between treated and untreated mice (Figure 4I). The data suggests less severe barrier impairment after therapeutic S1PR3 antagonism at earlier timepoints and confirms the absence of adverse treatment effects on the BBB.

### Antagonizing S1PR3 post-stroke affects astroglial glutamate receptor expression

**Figure 5:**
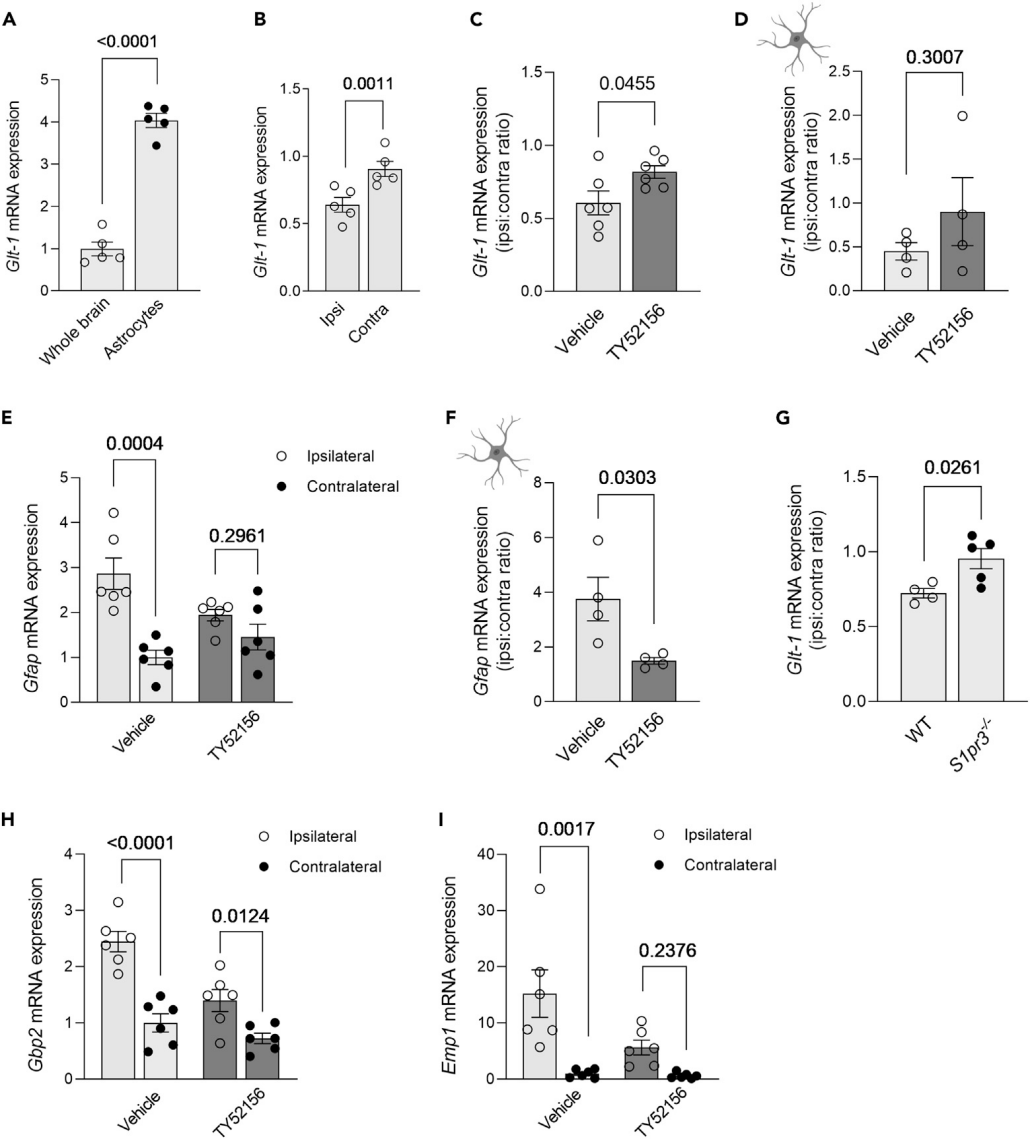
Antagonizing S1PR3 signaling induces neuroprotection 1-day post-tMCAo (A) *Glt-1* expression is enriched in astrocytes (*n* = 5) compared to whole brain tissue (*n* = 5). (B) Stroke decreases *Glt-1* expression in ipsilateral (*n* = 5) compared to the contralateral (*n* = 5) hemisphere. (C) Antagonizing S1PR3 with TY52156 (*n* = 6) increases ipsi:contra ratio of *Glt-1* expression compared to the vehicle controls (*n* = 6). (D) Antagonizing S1PR3 with TY52156 (*n* = 4) induces upregulation of astrocyte-specific ipsi:contra ratio of *Glt-1* expression compared to the vehicle group (*n* = 4). (E) Upregulation of *Gfap* expression in the ipsilateral hemisphere (*n* = 6) is diminished after S1PR3 antagonism with TY52156 (*n* = 6). (F) S1PR3 antagonism with TY52156 (*n* = 4) reduces *Gfap* expression compared to vehicle group (*n* = 4). (G) S1PR3 inhibitor-mediated neuroprotection induces higher ipsi:contra ratios for *Glt-1* expression in S1PR3^−/−^ (*n* = 5) compared to wild-type mice (*n* = 4). (H and I) Expression of astrocyte activation markers, (H) *Gbp-2* and (I) *Emp1*, are mitigated with administration of S1PR3 antagonist (*n* = 6) compared to vehicle group (*n* = 6). (A–D) and (F) and (G) are presented as mean ± SEM and were compared with a Student’s t test. (E), (H), and (I) are presented as mean ± SEM and compared with two-way ANOVA and Sidak post-hoc testing. Exact *p* values are given.

It has been shown that augmented S1P signaling inhibits astrocytic glutamate uptake in a dose-dependent manner.^36^^,^^37^ Astrocyte-mediated glutamate uptake is essential for neuroprotection during brain ischemia as it prevents extracellular glutamate concentrations from reaching excitotoxic levels.^38^ Glutamate transporter 1 (*Glt1*) expression, which is enriched in astrocytes compared to total brain tissue (Figure 5A), is reduced post-stroke^39^ (Figure 5B). Stroke associated GLT-1 alterations have been linked to increased neuroexcitotoxicity.^40^^,^^41^ Thus, increasing glutamate receptor expression has been applied to promote neuroprotection in experimental models.^42^ In this regard, unspecific S1PR modulation using fingolimod attenuated excitotoxicity and neuroinflammation.^43^ In our hands, specifically antagonizing S1PR3 at 4-h post-stroke mitigates the stroke-associated *Glt1* downregulation in the ipsilateral hemisphere, resulting in higher ipsi:contra *Glt1* expression ratios in the S1PR3 antagonist-treated compared to the vehicle group (Figures 5C and 5D). The concurrent changes in ipsilateral *Gfap* responses (i.e., attenuated stroke-associated increases in the S1PR3 antagonist group; Figures 5E and 5F) suggest an involvement of astrocytes in the S1PR3 antagonist-mediated neuroprotection. This is further supported by higher ipsi:contra ratios for *Glt1* expression in *S1pr3*^−/−^ compared to WT mice (Figure 5G) and the S1PR3 antagonist-associated alleviation of astrocyte activation marker expression (*Gbp2* and *Emp1*) in the ipsilateral hemisphere compared to the vehicle group (Figures 5H and 5I).

### Circulating S1PR3 levels are elevated in mice and patients with ischemic stroke

**Figure 6:**
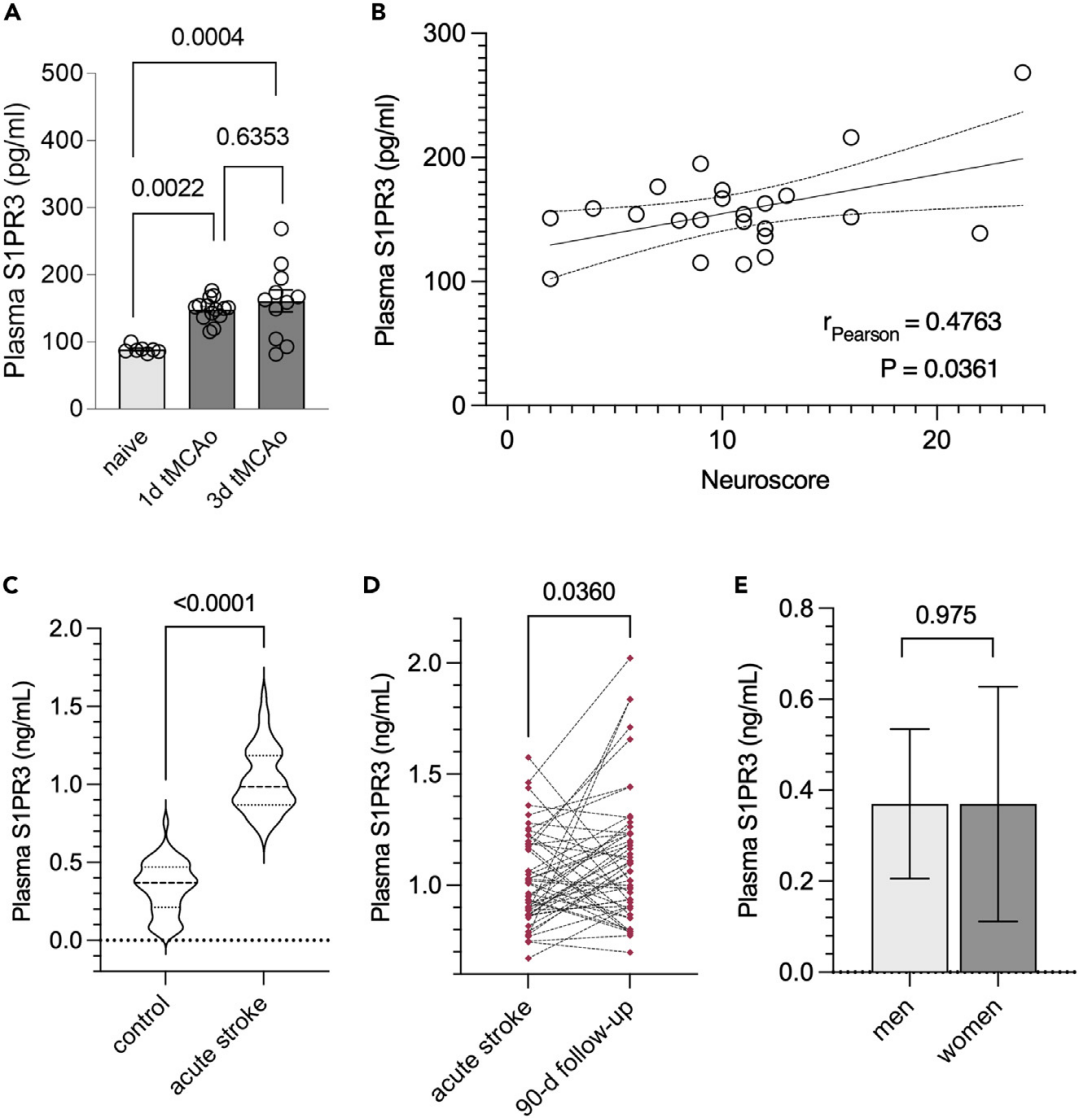
Circulating S1PR3 levels are increased in experimental and human ischemic stroke (A) Plasma S1PR3 concentrations are higher 1-day (*n* = 14) and 3-days (*n* = 11) post transient middle cerebral artery occlusion compared to naive mice (*n* = 8). (B) Pearson correlation showing significant associations between plasma S1PR3 levels and neuroscore post-stroke (*n* = 23). (C) Patients with acute ischemic stroke (*n* = 50) present with higher plasma S1PR3 concentrations compared to age- and sex-matched controls (*n* = 47). (D) Plasma S1PR3 concentrations are higher at 90-days follow-up compared to baseline (i.e., acute stroke) in patients with ischemic stroke (*n* = 50). (E) S1PR3 plasma levels are similar in men and women of the control group (*n* = 47). (A) is presented as mean ± SEM and is compared with a one-way ANOVA and Tukey’s post-hoc testing. (B) represents a Pearson correlation with two-tailed *p* value computation. (C) and (E) are presented as median ± interquartile range and are compared with Mann-Whitney. (D) is presented as before-and-after graph and is compared by Wilcoxon testing. Exact *p* values are given for all comparisons.

Inflammatory conditions can induce shedding of S1PR3 into the systemic circulation, allowing its quantification in plasma.^44^ Therefore, we tested whether the acute increase of cerebrovascular S1PR3 observed in our mouse models can also be detected in blood. Indeed, S1PR3 protein concentrations were elevated in the plasma of mice at 1-day and 3-days after tMCAo (Figure 6A). Pearson correlation with neuroscore revealed significant associations with S1PR3 plasma levels (Figure 6B), suggesting a link between S1PR3 plasma levels and stroke outcome in experimental stroke. To test whether a similar increase and association occurs in human stroke, we measured plasma S1PR3 concentrations in patients with acute ischemic stroke. We found higher S1PR3 levels in samples obtained from patients with acute ischemic stroke (*n* = 50) compared to age- and sex-matched controls (*n* = 47; Figure 6C; Table 1). Reexamination at 90-days post-stroke (*n* = 50) revealed generally increased S1PR3 plasma levels compared to baseline (i.e., acutely after stroke onset; Figure 6D). Spearman correlations between acute plasma S1PR3 and neurological function determined acutely post-stroke by National Institutes of Health Stroke Scale (NIHSS) showed no significant associations (Table 2). Similarly, functional status assessed at 90-days follow-up by modified Rankin Scale (mRS) was not correlated with acute S1PR3 plasma levels (Table 2). Additional exploratory correlation analyses between follow-up plasma S1PR3 and follow-up NIHSS or mRS showed no significant associations (Table 2). Because astrocytic S1PR3 signaling has been associated with proinflammatory responses,^45^^,^^46^^,^^47^^,^^48^ we tested a potential association between S1PR3 plasma levels and C-reactive protein (CRP) levels, as a surrogate marker of inflammation, in stroke patients during the acute phase post-stroke, but found no significant results (Table 2). To account for potential sex differences for S1P levels,^10^^,^^49^^,^^50^^,^^51^ we analyzed sex-specific differences of plasma S1PR3 levels in the control group but found no differences between men and women (Figure 6E). Together, these data suggest that changes in S1PR3 signaling in murine and human stroke are detectable in routine blood samples.

**Table 1:** Baseline characteristics human ischemic stroke cohort

	Control subjects *n* = 47	Stroke patients *n* = 50	*p*
Females, n (%)	24 (51%)	26 (52%)	ns^b^
Age (median ± IQR)	72.3 (66.7–79.6)	72.9 (64.4–78.7)	ns^a^
Hypertension, n (%)	23 (49%)	33 (66%)	ns^b^
Diabetes mellitus, n (%)^f^	4 (8%)	15 (30%)	0.008^b^
Hypercholesterolemia, n (%)	39 (85%)^c^	28 (56%)	0.002^b^
Current smoking, n (%)	1 (2%)	10 (20%)	0.006^b^
Ischemic heart disease, n (%)	6 (13%)	9 (18%)	ns^b^
BMI (median ± IQR)	25.3 (23.2–28.1)	25.6 (23.3–28.8)^d^	ns^a^
CRP (mg/L) (median ± IQR)	1.50 (0.63–3.38)^e^	1.85 (0.89–5.78)^e^	ns^a^
NIHSS baseline (median ± IQR)	NA	2.5 (1.0–4.2)	–
mRS day 90 (median ± IQR)	NA	1 (0–1)	–
NIHSS day 90 (median ± IQR)	NA	0 (0–1)^d^	–
Plasma S1PR3 (ng/mL)	0.37 (0.21–0.47)	0.99 (0.87–1.18)	<0.001

a Mann Whitney.

b Chi-square.

c One control subject missing data.

d One stroke patient missing data.

e 3 control subjects and 6 patients missing data.

f Definition diabetes: Fasting venous P-Glc ≥ 7 mmol/L, or fasting capillary P-Glc ≥ 7 mmol/L, or HbA1c ≥48, or previously known diet/oral/insulin treatment for diabetes.

**Table 2:** Correlations of plasma S1PR3 with clinical and laboratory parameters

	Correlation coefficient *n* = 50	*p*
**Acute plasma S1PR3 (ng/mL)**		

Acute NIHSS	0.019	0.894
Follow-up mRS	0.155	0.283
CRP (mg/L)	0.053^a^	0.735
Follow-up NIHSS	0.183	0.208

**Follow-up plasma S1PR3 (ng/mL)**		

Follow-up NIHSS	0.034^b^	0.819
Follow-up mRS	0.032	0.823

Correlation analysis with Spearman coefficients. CRP, C-reactive protein; mRS, modified Rankin Scale; NIHSS, National Institutes of Health Stroke Scale; S1PR3, sphingosine-1-phosphate receptor 3.

a 6 stroke patients missing data.

b One stroke patient missing data.

## Discussion

Modulation of S1PRs is a promising concept in cardiovascular disease, including ischemic stroke, but translatability to clinical trials has been hampered by the lack of spatiotemporal information on S1PR alterations. Here, we investigated how cerebrovascular S1PR3 expression is altered post-stroke and how genetic deletion or therapeutic antagonization of S1PR3 affects stroke outcome. Our experiments revealed an important role for S1PR3 signaling during the acute phase after stroke that was verified by functional improvement in the absence or after inhibition of S1PR3. We also determined the window for systemic pharmacological targeting of S1PR3 after stroke and showed that S1PR3 antagonist administration at 4-h post-stroke significantly improves stroke outcome, whereas later administrations appeared safe but had smaller effect sizes. Using cell type-specific methods, our results provide important evidence that astrocyte- but not endothelial-specific S1PR3 expression is the main if not sole driver of S1PR3 upregulation in acute ischemic stroke. An exploratory analysis of S1PR3 signaling in human stroke revealed higher circulating S1PR3 levels in acute ischemic stroke. Together, our results provide evidence for considering S1PR3 as a promising target for the development of new stroke therapies.

We confirm results from previously published analyses of S1PR1-5 expression profiles in whole brain tissue after tMCAo^20^^,^^21^ where S1PR3 rapidly increased within 24-h after stroke onset.^21^ Importantly, we also found that deletion of *S1pr3* resulted in functional improvement in response to tMCAo. These results contrast those of the hitherto only reported study using *S1pr3*^*−/−*^ mice in ischemic stroke,^52^ which used littermate controls to compare stroke outcome in response to pMCAo. We found stroke-induced S1PR3 elevation in pMCAo to be independent of reperfusion, suggesting that the contrasting findings are not due to the used stroke model. Since the characterization study for the *S1pr3*^*−/−*^ mouse indicates higher S1PR3 expression for fibroblasts isolated from WT mice compared to those isolated from *S1pr3*^*−/−*^ mice and WT littermate controls,^53^ it is likely that littermate control mice may carry altered S1PR3 expression in some cells, which could explain the differences observed for stroke outcome when using WT mice instead of littermates.

Pharmacologically antagonizing S1PR3 during the acute phase post-stroke significantly reduced infarct lesion size when administered immediately after reperfusion in a tMCAo model.^54^ Here, we provide evidence of infarct size reduction and cerebral blood flow (CBF) improvements in pMCAo, a model resembling stroke without reperfusion where treatment is given at more clinically relevant timepoints after stroke-onset. The observed CBF improvement after pharmacological S1PR3 inhibition may indicate potential vasoactive properties of S1PR3 modulation, in line with studies indicating that S1PR3 may modulate the vasoconstrictor response to S1P in cerebral blood vessels^55^ and systemic resistance vessels^56^ *ex vivo.* In more complex environments, S1P-induced regional blood flow reductions to the brain assessed with a laser doppler approach^55^ as well as lower coronary blood flow in response to exogenous S1P are abolished in the presence of S1PR3 inhibitor (i.e., pre-treatment).^18^ Importantly, our results that S1PR3 inhibition may antagonize BBB dysfunction suggest that the vasoactive effects may go beyond CBF modulation. *S1pr3* gradually increases between 3 and 6 h post-tMCAo followed by subsequent rapid rises beyond 6-h^21^ This temporal profile may explain our results showing no significant functional improvement when the S1PR3 antagonist was administered at 6- or 8-h post-stroke as S1PR3 levels may be too high for efficient antagonism. Importantly, none of the tested timepoints for systemic S1PR3 antagonization suggested adverse effects. Nonetheless, systemically targeting S1PR3 necessitates consideration of half-life, bioavailability, and undesirable effects on other organs. Ubiquitous S1PR expression makes systemic S1PR modulation particularly complicated, which is best exemplified by inconsistent results in clinical trials of the S1PR1 inhibitor fingolimod,^14^^,^^21^^,^^57^^,^^58^^,^^59^ which are further complicated by the fact that it targets more than one S1PR.^18^^,^^19^ Vascular S1PR1 that is crucial for maintaining vascular integrity^60^ decreases early after stroke onset,^20^^,^^61^ which can be exacerbated by fingolimod^56^ with implications for BBB integrity. Moreover, fingolimod also acts as an S1PR3 agonist,^45^^,^^62^ which may be detrimental considering our results and previously reported involvement of S1PR3 in astrocyte- and microglia-mediated inflammation,^45^^,^^46^^,^^47^^,^^48^ cerebrovascular responses^55^ to S1P or flow alterations or astrogliosis.^30^ Augmented S1P signaling has further been associated with alterations of astrocytic glutamate uptake,^36^^,^^37^ which may critically hamper neuroprotection during brain ischemia.^38^^,^^40^^,^^41^ The concept of post-stroke reduction of glutamate transporter expression^39^ has been successfully exploited to promote neuroprotection in experimental models.^42^ Excitotoxicity and neuroinflammation was previously dampened by unspecific S1PR modulation.^43^ Here, we provide first evidence that astrocytic S1PR3 links to *Glt1* alterations post-stroke and verify that specific S1PR3 antagonism may exert neuroprotection via specific S1P-S1PR signaling in astrocytes.

Despite strong evidence for increased S1PR3 expression post-stroke, data on cell-specific alterations in its expression and function are needed to target S1PR3 more efficiently and to circumvent potential side effects resulting from systemic modulation. In this regard, RNA sequencing of isolated middle cerebral arteries from male and female rats after tMCAo confirms the acute augmentation of vessel-associated *S1pr3* expression.^63^ Astrocyte-specific ipsilateral *S1pr3* upregulation post-stroke corroborates different *in vitro* studies, showing inflammation-induced increases of S1PR3 in astrocytes isolated from mice^44^^,^^45^^,^^46^^,^^47^ and humans.^64^ Further, S1PR3 was recognized as a marker of reactive astrocytes,^30^ verifying our data concentrating *S1pr3* expression along the ischemic lesion together with *Gfap*-positive astrocytes. Moreover, pharmacological antagonism that lowers astrocyte specific *Gfap* expression suggests a close link between S1PR3 and astrocyte activation. In contrast to its expression in astrocytes, acutely reduced endothelial *S1pr3* expression indicates an association with vascular damage at early stages during ischemia.^65^ The presence of *CD31*^+^ endothelial cells, especially in the damaged area, gradually increases 3-days after stroke^66^ reaffirming our data showing higher overall *S1pr3*^*+*^ *CD31*^*+*^ abundance in the ipsilateral hemisphere 3-days post-stroke. In contrast to astrocytes, *S1pr3* expression in the endothelium did not change up to 3-days post-stroke, which may also explain the lack of endothelial cell-specific *S1pr3* expression in our Ribotag experiments or overall lack of vascular protein response at the 3-days timepoint. Assessment of S1PR3 abundance per endothelial cells is warranted for later timepoints post-stroke (e.g., 7-days) to determine potential changes in endothelial-specific S1PR3 signaling at the time when angiogenesis occurs.^67^

For the first time to our knowledge, this study provides evidence of higher S1PR3 plasma levels in murine and human ischemic stroke and thus, supports findings obtained in a proteomic assessment of peripheral blood mononuclear cells (PBMCs) that identified S1PR3 as one of 146 core genes potentially related to ischemic stroke.^68^ Using receiver operating characteristic (ROC) analysis, the authors suggested S1PR3 as good ischemic stroke indicator.^68^ However, care must be taken when interpreting and generalizing these findings because of their small sample size (i.e., 20 stroke patients and 20 control subjects) without available information on sampling timepoint post-stroke, stroke severity or sex of the participating subjects. Although high S1PR3 levels in patients with acute stroke were not associated with neurological impairment, worse general outcome, or inflammation in our study, S1PR3 signaling may still be an interesting target to consider for further stroke therapy studies. As ELISA-based plasma S1PR3 detection does not allow for cell-specific source determination, it will be important to define its origin in future studies. M2 macrophages and PBMCs have been reported to have relatively higher S1PR3 expression in lymphoma models^69^ and lung cancer patients.^70^ Endothelial cells with direct interface to blood may also contribute to augmented S1PR3 shedding into the plasma.^44^ It remains to be determined whether alterations in their S1PR3 expression profiles are also evident in patients with ischemic stroke. Considering S1PR3’s spatiotemporal profile identified in murine stroke, it seems unlikely that endothelial S1PR3 is a major source of plasma S1PR3 augmentation in acute stroke. Relatively higher S1PR3 plasma levels detected at the 3-months follow-up, however, may well be due to higher endothelial cell-specific S1PR3 expression that could be associated with post-stroke angiogenesis.^67^ Although astrocytes do not directly intersect with the circulating blood, their significant contributions to rapid extracellular matrix disruption and tight junction degradation in response to ischemia^69^ may lead way to astrocyte-derived S1PR3 shedding to the blood.

Taken together, our study provides convincing evidence of involvement of S1PR3 in murine and human ischemic stroke and suggests a distinct spatiotemporal profile of S1PR3 expression within BBB constituents. Importantly, astroglial S1PR3 signaling associates with glutamate receptor expression and hence, may aid neuroprotection acutely post-stroke. Thus, cell-specific targeting should be explored as it might be of greater efficacy than systemic S1PR3 antagonism, which best attenuates stroke injury when applied early after stroke onset.

### Limitations of the study

Limitations associated with this study include the small sample size, the relatively short period for follow-up examinations and the mild stroke phenotype in the human cohort. Especially higher correlation coefficients obtained for associations between acute S1PR3 and follow-up NIHSS/mRS (compared to those obtained for comparisons with acute NIHSS/mRS) call for verification in larger stroke cohorts or cohorts including more severe stroke cases and additional clinical assessments. Moreover, the control cohort in this study was sampled approximately two years earlier than the patient samples. Since mouse plasma samples (both Sham and MCAo) were stored under similar conditions for up to three years before ELISA-based S1PR3 assessment, it is unlikely that protein degradation affected the results. Despite the acknowledged limitations, validation of stroke-associated increases in plasma S1PR3 levels in both mice and human already substantiates a potential pathophysiological importance of alterations in S1PR3 signaling post-stroke.

## STAR★Methods

### Key resources table

**Table:** 

REAGENT or RESOURCE	SOURCE	IDENTIFIER
**Antibodies**		

Goat polyclonal anti-GFAP	abcam	Cat#ab53554, RRID:AB_880202
Rabbit polyclonal anti-GFAP	Agilent	Cat#Z0334, RRID:AB_10013382
Rabbit polyclonal anti-S1Pr3	OriGene	Cat#TA329055
Mouse monoclonal anti-CD31 (clone P2B1)	abcam	Cat#ab24590, RRID:AB_448167
Rat monoclonal anti-CD13 (clone R3-63)	BioRad	Cat#MC2183, RRID:AB_323548
*Lycopersicon esculentum* (tomato) Lectin, DyLight488	Sigma-Aldrich	Cat#L0401
Cy5 AffiniPure Donkey Anti-Rat IgG	ImmunoResearch Laboratories	Cat#712-175-150, RRID:AB_2340671
Cy3 AffiniPure Donkey Anti-Rabbit IgG	ImmunoResearch Laboratories	Cat#711-165-152, RRID:AB_2307443
Goat anti-Rabbit IgG (H+L) Cross-Adsorbed Secondary Antibody, Alexa Fluor 405	Thermo Fisher Scientific	Cat#A31556, RRID:AB_221605
Goat anti-Mouse IgG (H+L) Highly Cross-Adsorbed Secondary Antibody, Alexa Fluor 594	Thermo Fisher Scientific	Cat#A11032, RRID:AB_2534091
Donkey anti-Rabbit IgG (H+L) Highly Cross-Adsorbed Secondary Antibody, Alexa Fluor 594	Thermo Fisher Scientific	Cat#A21207, RRID:AB_141637
Donkey Anti-Goat IgG H&L (Alexa Fluor 405)	abcam	Cat#ab175664, RRID:AB_2313502
Goat polyclonal anti-albumin	R&D systems	Cat#AF3329
Mouse monoclonal anti-b-tubulin	Sigma-Aldrich	Cat#T4026, RRID:AB_477577
Mouse monoclonal anti-b-actin (clone C4)	Merck	Cat#MAB1501, RRID:AB_2223041
Goat anti-rabbit, HRP-linked	Cell Signaling Technology	Cat#7074S, RRID:AB_2099233
Peroxidase-AffiniPure Goat Anti-Mouse	Dianova	Cat#115-035-062, RRID:AB_2338504
anti-HA antibody (12CA5)	Sigma-Aldrich	Cat#11583816001

**Biological samples**		

Plasma samples from human subjects	Regional Ethical Review Board in Lund, Sweden (Registration No. 2016/179)	Swedish Ethical Review Authority (Registration No. 2020-07047)

**Chemicals, peptides, and recombinant proteins**		

TRIzol™ Reagent	Thermo Fisher Scientific	Cat#15596018
Blocking Reagent	Roche	Cat#11096176001
Fluoromount-G Mounting Medium	Thermo Fisher Scientific	Cat#00-4958-02
Fluoromount-G Mounting Medium with DAPI	Thermo Fisher Scientific	Cat#00-4959-52
SuperSignal West femto Maximum Sensitivity Substrate	Thermo Fisher Scientific	Cat#34095
Pierce™ BCA Protein Assay Kits	Thermo Fisher Scientific	Cat#23227
TransBlot Turbo Mini-size LF PVDF membrane	BioRad	Cat#10026934
2,3,5-Triphenyltetrazolium chloride (TTC)	Sigma-Aldrich	Cat#93410, CAS: 298-96-4
Superfrost Plus Microscope Slides	VWR European	Cat#631-0108
Formaldehyde, 37% w/w aq. soln., stab. with 7-8% methanol	Thermo Fisher Scientific	Cat#A16163, CAS:50-00-0
High-Capacity cDNA Reverse Transcription Kit	Thermo Fisher Scientific	Cat#4368814
RNeasy Micro Kit	Qiagen	Cat#74004
RNeasy® Plus Mini Kit	Qiagen	Cat#74134
QIAzol	Qiagen	Cat#79306
Protein G Dynabeads	Life Technologies	Cat#10004D
RNA 6000 pico Kit	Agilent Technologies	Cat#5067-1513
dextran ∼70,000	Sigma-Aldrich	Cat#31390, CAS: 9004-54-0
1x protease inhibitor	Thermo Fisher Scientific	Cat#87785
1x phosphatase inhibitors	Thermo Fisher Scientific	Cat#78420
Tamoxifen	Sigma-Aldrich	Cat#T5648, CAS: 10540-29-1
CAY10444	Cayman	Cat#10005033; CAS: 298186-80-8
TY52165	Biomol	Cat#Cay19119-5; CAS: 934369-14-9
Buprenorphine, TEMGESIC Ampullen 0.3 mg Injektionslösung	Reckitt Benckiser Healthcare	Cat#003459288 (PZN)
PrimaQUANT 2x CYBR Blue	Steinbrenner-Laborsysteme	Cat#SL-9912B
SYBR Green PCR Master Mix	Thermo Fisher Scientific	Cat#4369702
Isoflurane, Vetflurane®	Virbac	Cat#VNR137317
TWEEN® 80	Sigma	Cat#P4780; CAS: 9005-65-6

**Critical commercial assays**		

S1PR3 Elisa Kit Mouse	Nordic Biosite	Cat# EKX-UXD4XY-96
S1PR3 Elisa Kit Human	Nordic Biosite	Cat# OKEH04291-96
Multiplex fluorescent RNAscope	Advanced Cell Diagnostics	Cat#320850

**Experimental models: Organisms/strains**		

C57BL/6N mice	Charles River, Germany or Taconic, Denmark	N/A
*Cnx43*^Cre-ER(T)^/RiboTag mice	Rakers et al.^29^	N/A
*Cdh5*^Cre-ER(T)^/RiboTag mice	Jambusaria et al.^71^	N/A
*S1pr3*^-/-^ mice	Ishii et al.^53^, provided by Jerold Chun	N/A
Rpl22^tm1.1Psam^ RiboTag mice	Sanz et al.^72^	N/A

**Oligonucleotides**		

qPCR - S1PR3 - 5’ ->3’ Fw: CAAGCCTAGCGGGAGAGAAA; Rev: ACTGCGGGAAGAGTGTTGAA	Eurofins Genomics	N/A
qPCR - Gfap - 5’ ->3’ Fw: AAGGTCCGCTTCCTGGAA; Rev: GGCTCGAAGCTGGTTCAGTT	Eurofins Genomics	N/A
qPCR - Glt-1- 5’ ->3’ Fw: CCGACCGTATAAAATGAGCTACC; Rev: ATTCCTGTGACGAGACTGGAG	Eurofins Genomics	N/A
qPCR - Gbp2- 5’ ->3’ Fw: TGCTGGATCTTTGCTTTGGC; Rev: TTAGCGGAATCGTCTACCCC	Eurofins Genomics	N/A
qPCR - Emp1- 5’ ->3’ Fw: CTGTTTGTCTCCACCATTGCC; Rev: ACCACCAGTGCAGTTCTTCC	Eurofins Genomics	N/A
qPCR - L14 - 5’ ->3’ Fw: GGCTTTAGTGGATGGACCCT; Rev: ATTGATATCCGCCTTCTCCC	Eurofins Genomics	N/A
RNAscope probe *S1pr3* (ACD)	Bio-Techne	Cat#435951
RNAscope probe *CD31* (ACD)	Bio-Techne	Cat# 316721
RNAscope probe Sox9 (ACD)	Bio-Techne	Cat#401051
RNAscope probe *Gfap* (ACD)	Bio-Techne	Cat#313211
Positive control probe (ACD)	Bio-techne	Cat#32088
Negative control probe	Bio-Techne	Cat#320871

**Software and algorithms**		

NIS Elements	Nikon	RRID:SCR_014329
Leica LAS-X	Leica	RRID:SCR_013673
Fiji/ImageJ	ImageJ	https://imagej.net, RRID:SCR_003070
GraphPad Prism (Version 9.2.0)	GraphPad Software	https://www.graphpad.com/, RRID:SCR_002798
Matlab	Mathworks	RRID:SCR_001622
Paravision 6.0.1	Bruker	Version 6.0.1, RRID:SCR_001964
SPSS	IBM Corp.	Version 28, RRID:SCR_002865
CellProfiler Image Analysis Software (Version 3.1.9.)		Version 3.1.9, RRID:SCR_007358
Biorender		RRID:SCR_018361, Institutional license EMV, Lund University, Sweden & DZNE Bonn, Germany

**Other**		

A1RHD confocal microscope	Nikon	https://www.microscope.healthcare.nikon.com/
DMI600B	Leica	https://www.leica-microsystems.com/
ChemiDoc MP	Bio-Rad	Cat#12003154
Trans-Blot Turbo Transfer System	Bio-Rad	Cat #1704150
C1000 Touch Thermal Cycler	Bio-Rad	Cat#1855484
AxioScan.Z1	Zeiss	https://www.zeiss.com/microscopy/
LSM900	Zeiss	https://www.zeiss.com/microscopy/
9.4 T MRI scanner with Bruker BioSpec AVIII electronics	Bruker	https://www.bruker.com/
Laser Doppler	Moor Instrument	https://www.moor.co.uk/
Precellys®24	Bertin-Instruments	Cat# P002391-P24T0-A.0
UltraTurrax TP18-10	Janke & Kunkel KG	N/A
Precast NuPAGE™ gel	Thermo Fisher Scientifc	Cat# WG1402BOX
Stella imaging system	Bio-Imaging	N/A (discontinued)
pluriStrainer Mini 20 μm	Pluriselect	Cat#43-10020-40

### Resource availability

#### Lead contact

Further information and requests for resources and reagents should be directed to and will be fulfilled by Anja Meissner (anja.meissner@med.lu.se or anja.meissner@uni-a.de).

#### Materials availability

No unique reagents were generated in this study.

#### Data and code availability

• All data reported in this paper will be shared by the lead contact upon request.
• This paper does not report original code.
• Any additional information required to reanalyze the data reported in this paper is available from the lead contact upon request.

### Experimental model and study participant details

#### Research animals

All animal experiments presented in this study were approved by LANUV of North Rhine-Westphalia (AZ #81-02.04.2019.A214) and by the institutional ethics committee at Lund University (Dnr.5.8.18 – 08160/2021) and were conducted in accordance with ARRIVE guidelines and European animal protection laws (Directive 2010/63/EU). Adult male mice between 3 - 5 months of age were housed under 12/12 hours light-dark cycle with access to food and water *ad libitum* and kept under specific pathogen-free conditions. Wild-type (WT) C57BL/6N were purchased from Charles River (Sulzfeld, Germany) or Taconic (Ejby, Denmark). *S1pr3*^-/-^ mice were kindly provided by Jerold Chun^53^ and bred in a conventional animal facility under standard conditions. RiboTag Rpl22^tm1.1Psam^ mice^72^ were crossbred with astrocyte-(*Cnx43*) or endothelial-specific (*Cdh5*) Cre recombinase mice to generate *Cnx43*^Cre-ER(T)^/RiboTag^29^ and *Cdh5*^Cre-ER(T)^/RiboTag^71^ mice, respectively. Following tamoxifen-induced recombination at 10 weeks of age (100 mg/kg Tamoxifen (Sigma-Aldrich #T5648) dissolved in 100 % ethanol and sunflower oil to the final concentration of 20 mg/ml was injected intraperitoneally for five consecutive days), HA-tagged *Rpl22* was specifically expressed in astrocytes or endothelial cells. To investigate the mechanisms of astrocyte or endothelial cell-specific responses to ischemic stroke, mice were subjected to transient middle cerebral artery occlusion (tMCAo) three weeks after the last tamoxifen injection. At the end of the experiment, mice were euthanized using an overdose of inhalation anesthesia (Vetflurane, Virbac) and subsequent transcardiac perfusion with saline.

#### Human cohort

All human investigations conformed to the principles outlined in the Declaration of Helsinki. Plasma samples from subjects included in this study were selected from the Lund Stroke Recovery Study, which is a sub-cohort of the Lund Stroke Register^73^ that includes first-ever stroke incidents occurring in citizens of eight municipalities in southern Sweden. The study was approved by the Regional Ethical Review Board in Lund, Sweden (Registration No. 2016/179) and the Swedish Ethical Review Authority (Registration No. 2020-07047). Participants (51% females; median age 72 years) gave informed consent prior to the inclusion of people in the study. Based on *a priori* power calculations, 50 patients from the Lund Stroke Recovery Study who experienced a first ever ischemic stroke during 2021 were selected and age- and sex-matched with control subjects collected during 2018-2019. Plasma samples from stroke patients (N=50, with 52% females) taken acutely after stroke event (i.e., within 11-days; median 56-hours) and at a 90-days follow-up and from control subjects (N=47 with 51% females, baseline characteristics of cohort see Table 1) were extracted from the regional Biobank (Biobank Sverige, Södra Sjukvårdsregionen).

### Method details

**Transient middle cerebral artery occlusion (tMCAo)** in mice was performed as previously described.^74^^,^^75^ Mice were subcutaneously injected with Buprenorphine (0.05 mg/kg; Reckitt Benckiser Healthcare) 30 min before the surgical procedure. Eyes were protected from drying with eye ointment (Bepanthen, Bayer). Inhalation anesthesia was initiated using 3 % Isofluorane (Vetflurane, Virbac) with a mixture of 30 % O_2_ and 70 % N_2_O and reduced to 1.5 – 2 % for the surgery. The body temperature was kept at 37 ± 0.5°C using a closed-loop controlled rectal probe and an electric blanket (CODA®Monitor; Kent Scientific). The mouse was placed in a prone position, the skin on the head was disinfected with Octenisept® (Schülke & Mayr) and locally anesthetized with 1 % xylocain (Dentsply Sirona). A 1 cm long skin incision was made from the superior nuchal line to the nasion to expose the skull. A laser Doppler plastic fiber probe was attached perpendicularly to the skull in a small hole (∼ 2 mm) drilled directly above the left middle cerebral artery (MCA). Cerebral blood flow was monitored during the surgery with the laser Doppler device (Moor Instrument). Afterwards, the mouse was turned to a supine position and 1 mm transverse neck incision was made. The large pair of salivary glands were separated from each other and placed to the side. The common carotid artery (CCA), internal carotid artery (ICA) and external carotid artery (ECA) were identified and dissected from surrounding connective and fatty tissue. The CCA was carefully separated from the vagus nerve and temporarily occluded using vascular suture (7/0; Suprama). The distal part of the ECA was permanently occluded and another loose suture was prepared close to the bifurcation. The ICA was closed with a vascular clip. The silicon coated monofilament (9-10 mm coating length, 0.19 ± 0.01 mm tip diameter; Doccol) was inserted to the small incision in ECA and secured by tightening the vascular suture. The ECA was cut between the two sutures, the vascular clip was removed from the ICA and the monofilament was advanced through the ICA until it reached the MCA, which was confirmed by the drop of the regional blood flow (> 75 % of the baseline values) monitored by laser Doppler flowmetry. The reperfusion was induced by removing the monofilament after 60 minutes. The ECA was permanently closed and the CCA suture was removed. The reperfusion had to reach >75 % of the baseline values to include the mouse in the study. The neck and head incision were closed with a silk suture (Braun). 100 μl of saline was injected intraperitoneally to prevent dehydration. Mice were placed in a recovery chamber set to 37°C. Sham surgeries were performed using the same protocol without monofilament insertion. Post-surgery analgesia using Buprenorphine (0.05 mg/kg; Reckitt Benckiser Healthcare) was subcutaneously administered every 12-hours for up to 2-days.

#### Permanent MCAo (pMCAo)

C57Bl6 mice were anesthetized using 3 % Isoflurane (IsoFlo® vet 100 %) with a mixture of 30 % O_2_ and 70 % N_2_O and reduced to 1.5 – 2 % for the surgery. The body temperature was kept at 37 ± 0.5°C using a heating pad. The mouse was placed on the side and 1 cm incision was made between the left orbit and the external auditory meatus. Using electrocoagulation forceps set at 12 W, the temporal muscle was detached from the skull. 1 – 2 mm area of the skull above the MCA was thinned with the dental drill until the part of the skull was possible to remove. The MCA was permanently coagulated with electrocoagulation forceps set at 7 W proximal to the bifurcation followed by transection of the artery to ensure the occlusion. The temporal muscle was placed back to its original position and the incision was sutured. Mice were placed under the infrared lamp to recover from the anesthesia. Post-surgery analgesia using Buprenorphine (0.05 mg/kg; Reckitt Benckiser Healthcare) was administered every 12-hours for up to 2-days.

#### Preparation and administration of S1PR3 antagonist

S1PR3 antagonists CAY10444 (Cayman, 10005033) or TY52165 (Biomol, Cay19119-5) were dissolved in 0.375 % Tween 80 and injected at a dose of 1 mg/kg intraperitoneally in 100 μl of saline at 4-, 6- or 8-hours after MCAo. Vehicle solution (100 μl 0.375 % Tween 80) was administered in respective control mice at 4-, 6- or 8-hours after MCAo.

#### Neuroscore

Neurological function was evaluated 1- and 3-days after tMCAo using an extended scoring system.^26^ The sum of general and focal deficits ranged between 0 (no deficit) and 56 (the poorest performance in all categories).

#### Brain tissue preparation for molecular studies

At day 1- or 3-days after MCAo, mouse brains were transcardially perfused with phosphate buffer saline (PBS; 137 mM NaCl, 2.7 mM KCl, 10 mM Na_2_HPO_4_, 1.8 mM KH_2_PO_4_). The olfactory bulbs and cerebellum were removed, and the brains were separated into the ipsilateral (ischemic) and contralateral hemispheres and processed separately. Brain tissue was homogenized in 1 ml PBS with ceramic beads using the tissue homogenizer Precellys®24 or an UltraTurrax TP18-10 (Janke & Kunkel KG) and stored at –80°C for further processing.

#### Real-time quantitative polymerase chain reaction (RT-qPCR)

Total RNA was extracted from brain homogenates using TRIzol Reagent (Thermo Fisher Scientific, 15596018) according to manufacturer’s instructions. RNeasy® Plus Mini Kit (Qiagen, 74134) was used to purify RNA from the aqueous phase and RNA was eluted using 60 μl of RNase-free water. 500 ng of RNA was reversely transcribed to cDNA using High-Capacity cDNA reverse Transcription Kit (Thermo Fisher Scientific, 4368814). Gene expression (see key resources table for primer pairs used in qPCR) was detected using PrimaQUANT 2x CYBR Blue (Steinbrenner-Laborsysteme, SL-9912B) or SYBR Green PCR Master Mix (Thermo Fisher Scientific, #4369702) and C1000 Touch Thermal Cycler (Bio-Rad). All samples were run in triplicates and the relative gene expression was calculated from the standard curve and normalized to the housekeeping gene L14.

#### Immunoprecipitation of mRNA with RiboTag

Each brain hemisphere from *Cnx43*^Cre-ER(T)^/RiboTag or *Cdh5*^Cre-ER(T)^/RiboTag was homogenized in 1 ml polysome buffer (PSB; 50mM Tris pH 7.5, 100 mM KCl, 12 mM MgCl_2_, 1% Nonidet P-40, 1mM Dithiothreitol, 3.75 μl/ml RNase inhibitor, 100 μg/ml Cycloheximide, 2x Protease inhibitor, 1x Phosphatase inhibitor) using Precellys®24. Supernatant 1 (S1) was prepared by centrifugation at 10,000 g for 10 min at 4°C. Total mRNA was used as control and prepared by mixing 100 μl of S1 with 700 μl QIAzol (Qiagen, 79306) to subsequently extract mRNA. The rest of S1 was pre-cleared by incubation with Protein G Dynabeads (PGDB; Life Technologies, 10004D) for 30 min at 4°C. Pre-cleared S1 was transferred to a tube containing anti-HA antibody (12CA5; Sigma-Aldrich, 11583816001) and incubated for 45 min at 4°C. The lysate with the antibody was then added to a new volume of PGDB and incubated for 80 min at 4°C. In the last step, samples were placed on the magnetic rack to allow PGDB to adhere to the wall and the unbound fraction was discarded. PGDB were washed three times with high salt buffer (HSB; 50mM Tris pH 7.5, 300 mM KCl, 12 mM MgCl_2_, 1% Nonidet P-40, 1mM Dithiothreitol, 1.25 μl/ml RNase inhibitor, 10 μg/ml Cycloheximide, 0.5x Protease inhibitor, 1x Phosphatase inhibitor) followed by additional three washes with extra high salt buffer (EHSB; 50mM Tris pH 7.5, 300 mM KCl, 300mM NaCl, 12 mM MgCl_2_, 1% Nonidet P-40, 1mM Dithiothreitol, 1.25 μl/ml RNase inhibitor, 10 μg/ml Cycloheximide, 2x Protease inhibitor, 1x Phosphatase inhibitor) to reduce the background. Actively translated mRNA was extracted with QIAzol and RNeasy Micro Kit (Qiagen, 74004) according to the manufacturer’s instructions. In the last step, mRNA was eluted with 28 μl of RNase-free water. RNA quality was evaluated using Agilent RNA 6000 pico Kit (Agilent Technologies, 5067-1513) on an Agilent 2100 Bioanalyzer. To avoid degradation, mRNA was directly transcribed to cDNA and stored at -20°C.

#### Multiplex fluorescent RNA scope

Fresh frozen brains were sectioned (coronal) at 20 μm thickness and used for Multiplex fluorescent RNAscope (Advanced Cell Diagnostics, 320850) following the manufacturer’s protocol for fresh frozen tissue using probes against *S1pr3* (ACD, Bio-Techne, 435951), *CD31* (ACD, Bio-Techne, 316721), Sox9 (ACD, Bio-Techne, 401051) and *Gfap* (ACD, Bio-Techne, 313211). A positive control probe (ACD, Bio-techne, 32088) was used to assess RNA Integrity of used sections. Negative control probe (Bio-Techne, 320871) served for determination of background fluorescence. Probes were labeled with fluorophores, Atto488 – C3, Atto 550 – C1, Atto 647 – C2. Whole brain slices were first imaged using a slide scanning microscope AxioScan.Z1, 20x objective followed by imaging with Zeiss LSM900 microscope, 40x oil objective with z-stack. For detection of *S1pr3*/*Sox9*/*Gfap* colocalization, z-stacks of 4 μm with a z-slide interval of 0.19 μm were imaged. Thirty images per hemisphere were sampled from each brain slice. Z-stacks were processed by calculating the maximum intensity projections using Zen software. Semi-automated analysis pipeline was designed in CellProfiler (version 3.1.9.) and used to count mRNAs visualized by respective probes. Primary objects were identified based on DAPI (staining cell nuclei) with a diameter range of 70-300 pixels units. Each primary object was expanded by 80 pixels units. From the list of detected cells with a related numbers of dots per channel were identified cells positive for *S1pr3* defined with ≥ 5 mRNAs/cell, *Sox9* and *Gfap* with ≥ 8 mRNAs/cell.

#### Immunohistochemistry

Following transcardiac perfusion with saline, brains were immediately removed and post-fixed in 4 % PFA for 24 h at 4°C, cryoprotected in 15-30 % gradient of sucrose in PBS and stored at 4°C until sectioning. Ten μm cryostat-sectioned brain slices were fitted onto Superfrost Plus glass slides, washed with PBS and incubated for 30 min at room temperature with blocking buffer, followed by overnight incubation with primary antibodies. Primary antibodies used were GFAP (1:500 goat, abcam), S1Pr3 (1:200, rabbit, OriGene), CD31 (1:200, mouse, abcam), CD13 (1:300, rat, BioRad) and Lectin from *Lycopersicon esculentum* labelled with DyLight 488 (1:200, Sigma). For CD31 staining, slides were pre-incubated for 7 min in PBS containing 1 % SDS at RT to allow antigen retrieval, followed by washing in PBS and blocking as described above. After washing in PBS, sections were incubated with appropriate Alexa Fluor conjugated secondary antibodies for 1 h at RT (1:500-1:1,000 dilutions, see key resources table; Thermo Fisher Scientific or Jackson ImmunoResearch Laboratories). Sections were washed and mounted with Fluoromount-G Mounting Medium or Fluoromount-G Mounting Medium with DAPI, and examined under a DMI6000B (Leica, Wetzlar, Germany). Images were acquired with Leica LAS X, and then processed in ImageJ (https://imagej.net; version.1.54f). Quantification of colocalization of GFAP^+^, CD31^+^ and CD13^+^ cells with S1PR3 and vessels fluorescently labelled with Lectin were done by manual counting using 325x250 μm stacks of 10 μm thick. Five regions of interest in 5 different images per animal were counted. Representative images were visualized under a Nikon A1RHD confocal microscope with a CFI Plan Apochromat Lambda 20x/40x NA 0.75 (Nikon Instruments Inc., Tokyo, Japan) and then acquired with NIS-elements (Laboratory Imaging, Nikon), and processed in ImageJ (https://imagej.net; version 1.54f). Representative confocal images were denoised using the Nikon’s artificial intelligence denoising algorithm (Denoise.ai) in NIS-elements.

#### Separation of brain tissue into vessel-rich and vessel-depleted samples

Brain tissue fractionation was performed as described previously.^27^ Briefly, each hemisphere was homogenized in 1 ml B1 (HBSS with 10 mM HEPES) with a 21 G cannula mounted on a syringe and centrifuged at 2,000 g for 10 min at 4°C. The supernatant representing the vessel-depleted brain fraction was mixed with equal volume of 2x RIPA buffer (20 mM Tris pH 8.0, 2 mM EDTA, 2 % Triton X-100, 0.2 % sodium deoxycholate, 0.2 % SDS, 280 mM NaCl) supplemented with 1x protease (Thermo Fisher Scientific, 87785) and 1x phosphatase inhibitors (Thermo Fisher Scientific, 78420). Protein was isolated as described below in Western blot section. Pellets containing vessel-rich fractions were resuspended with B2 (B1 with 18 % dextran ∼70,000; Sigma Aldrich, 31390), mixed thoroughly and centrifuged at 4,400 g for 15 min at 4°C. Pellets were then resuspended in B3 (B1 with 1 % BSA) and transferred onto a 20 μm cell strainer (Pluriselect, 43-10020) followed by centrifugation at 200 g for 1 min at 4°C to collect the vessel-rich fraction. The vessel-rich fraction captured on the strainer was washed twice with B3 and subsequent centrifugation. The vessel-rich fraction was collected with B3 and pelleted by centrifugation at 2,000 g for 5 min at 4°C. Finally, the vessel-rich fraction was washed with B1 to remove BSA. Purified vessel-rich fractions were lysed with 1x RIPA buffer supplemented with protease and phosphatase inhibitors and homogenized in a glass micro homogenizer (Radnoti). The lysate was vigorously vortexed and incubated for 30 min on ice. The insoluble material was removed by centrifugation at 20,000 g for 10 min at 4°C and samples were stored for subsequent Western blotting.

#### Western blot

Tissue homogenates were solubilized in lysis buffer (10 mM Tris pH 8.0, 1 mM EDTA, 1 % Triton-X, 0.1 % sodium-deoxycholate, 0.1 % SDS, 140 mM NaCl) supplemented with phosphatase (Thermo Fisher Scientific, 78420) and protease inhibitors (Thermo Fisher Scientific, 87785) for 30 min on ice. To remove insoluble material, protein extracts were centrifuged for 10 min at 20,000 g at 4°C. Protein concentration was measured using Pierce™ BCA Protein Assay Kit (Thermo Fisher Scientific, 23227). A defined concentration of protein was loaded on 4-12 % Precast NuPAGE™ gel (Thermo Fisher Scientifc, WG1402BOX) and separated at 100 V for 2.5-hours. Proteins were transferred onto PVDF membranes (GE Healthcare and Bio-Rad) using a semi-dry blotter or a Trans-Blot Turbo Transfer System (Bio-Rad). Membranes were blocked in 5 % non-fat dry milk for 1 h at room temperature (RT) followed by incubation with primary antibody anti-S1PR3 (1:2,000, OriGene, TA329055), anti-albumin (1:1,000, R&D systems, AF3329), anti-GFAP (1:1,000, Agilent, Z0334) anti-β-tubulin (1:5,000, Sigma-Aldrich, T4026) and anti-β-actin (1:5,000, Merck, MAB1501) overnight at 4°C. Next, membranes were incubated with HRP-conjugated goat anti-rabbit (1:10,000, Cell Signaling Technology, 7074S) or goat anti-mouse (1:10,000, Dianova, 115-035-062) antibody for 2-hours at RT. Proteins were visualized by enhanced chemiluminescence (SuperSignal™ West femto Maximum Sensitivity Substrate Thermo Fisher Scientific, 34095) using a Stella imaging system (Bio-Imaging) or a ChemiDoc MP (Bio-Rad). Relative S1PR3, GFAP and serum-albumin protein expression normalized to β-actin or β-tubulin was analyzed using ImageJ (https://imagej.net; version 3.7.4).

#### Magnetic resonance imaging (MRI)

Imaging was performed on a preclinical 9.4 T MRI scanner with Bruker BioSpec AVIII electronics (Bruker) operating with ParaVision 6.0.1 and a gradient strength of 670 mT/m. The coils used were a quadrature volume resonator (112/087) for transmission and a mouse brain 2x2 phased array coil for reception (Bruker). Mice were anesthetized with 3.5 % isoflurane with a mixture of NO_2_/O_2_ (1:1) and respiration was kept between 65–90 breaths with 1.5-2.5 % isoflurane during the imaging. The head was fixed with a tooth bar. Additionally, mice were covered with a heating blanket to ensure constant temperature between 36°C–37°C. The body temperature and respiration rate were controlled with a SA Instrument (Stony Brook) monitoring system throughout the whole imaging time. T2-weighted images of the whole brain were acquired using Rapid Imaging with Refocused Echoes (RARE) sequence with repetition time = 3.4 s, echo time = 33 ms, 30 slices with 0.5 mm thickness, resolution of 100x100 μm^2^, field of view 20x12 mm^2^ and 13 averages. To assess CBF, arterial spin labeling (ASL) was utilized with a Look-Locker (LL) FAIR TrueFISP (repetition time = 20 s, echo time = 1.2 ms and acquisition time = 127.2 ms).^76^ Three to four coronal slices were imaged per mouse and thirty inversion-recovery points were sampled over 7.63 s. The resolution was 233x234 μm^2^, with field of view 17x15 μm^2^, slice thickness 2 mm and 32 repetitions. Images from LL TrueFISP were pre-processed using MATLAB based on previously published protocols.^76^

#### Infarct size determination using 2,3,5-Triphenyltetrazolium chloride (TTC)

Perfused brains were cut into 1 mm coronal slices using a brain matrix and incubated for 20 min at 37°C in 2 % TTC (Sigma-Aldrich, 93140) dissolved in saline. Brain sections were digitalized, and the infarct lesion size was analyzed with ImageJ and presented as a percentage of the contralateral hemisphere.

#### Plasma S1PR3 ELISA

Prior to transcardial perfusion, blood was collected from vena cava using EDTA coated tubes to prevent coagulation. Plasma was separated by centrifugation at 1,000 g for 10 min at RT and used for determination of S1PR3 concentration using a commercially available S1PR3 ELISA kit (Nordic BioSite, EKX-UXD4XY-96) as per manufacturer’s instructions.

In human samples, EDTA plasma was extracted from Region Skåne Biobank and S1PR3 concentration was determined using a commercially available S1PR3 ELISA kit (Nordic BioSite, OKEH04291-96) as per manufacturer’s instructions.

#### Clinical and laboratory assessments of human study participants

Participants’ height (cm) and weight (kg) were measured, and body mass index (BMI) was calculated. Resting blood pressure (mmHg) was measured. Current smoking (yes/no), and anti-hypertensive treatment (AHT) were self-reported or collected from medical files. Hypertension was defined as BP >=140/90 or AHT. Diabetes mellitus was defined as either self-reported treatment for diabetes mellitus (diet or use of anti-diabetic medication), HbA1c >=48 mmol/mol or fasting plasma glucose ≥7.0 mmol/L. Hypercholesterolemia was defined as total cholesterol >5 mmol/L, LDL cholesterol > 3mmol/L or use of lipid-lowering medication (any of statin, bezafibrate, cholestyramine, ezetimibe treatment).

Each patient’s neurological status was assessed with the National Institutes of Health Stroke Scale (NIHSS).^77^ Functional status at 3 months was assessed with the Modified Rankin Score (mRS)^78^ on a scale between 0 (no residual symptoms) to 6 (death).

Blood samples were drawn and analyzed for plasma glucose, C-reactive protein (CRP), total serum cholesterol, high-density lipoprotein (HDL), and low-density lipoprotein (LDL), using standard clinical methods at the Department of Clinical Chemistry, Skåne University Hospital Lund/Malmö, which is part of a national standardization and quality control system.

### Quantification and statistical analysis

#### Analysis of CBF in experimental stroke

Images from LL TrueFISP were pre-processed in MATLAB (Mathworks, USA) using an analysis pipeline previously published.^76^ Pre-processed images were then further analyzed in ImageJ (https://imagej.net) by drawing ROI around the whole brain. Corresponding CBF values are presented as ml/100 g/min.

#### Analysis of infarct lesion in experimental stroke

Infarct lesion was analyzed in ImageJ (https://imagej.net) by manually drawing region of interest (ROI) around the infarct lesion and contralateral hemisphere. The size of the infarct lesion was presented as the percentage of the contralateral hemisphere (corrected for edema).

#### Analysis of areas of infarct lesion with different level of damage in experimental stroke

To analyze areas with different water content (Figure S9), first the outside area was cleared, and the resulting image was saved as *tiff* file. Using ImageJ, the threshold for brightness/contrast of all images was set to 128. All the visibly bright areas other than the infarct lesion were removed and data files with numbers of pixels for each value based on the corresponding histogram 0 – 255 /per each set of images were generated.

Next, the value preceding the first value with 0 pixels was identified and used as a threshold. Ascending numbers starting with 1 were assigned starting with pixel 128 until the pixel representing the threshold. 80 % and 20 % of the last assigned number was calculated and values in the range 80 % - 20 % were summed up and used as peri-infarct area that was presented as percentage of total infarct.

#### Statistical analysis of data generated in experimental stroke models

Statistical analysis was performed in GraphPad Prism (version 9.2.0). All data sets were tested for normal Gaussian distribution using Shapiro-Wilk normality test. To test differences between two groups Student t-test, Mann-Whitney or Wilcoxon matched-pairs tests were used. One-way ANOVA followed by Tukey’s post-hoc testing was used to test differences between multiple independent groups. Repeated measures two-way ANOVA followed by Sidak’s multiple comparison test were used to compare multiple groups defined by two factors. Pearson correlation analysis was carried out with two-tailed significance testing and computation of exact correlation coefficient (Pearson’s r). Normally distributed data are presented as mean ± standard error of the mean (SEM). Data that are not normally distributed are presented as median ± interquartile range. For all data sets, N represents the number of animals. Differences were considered significant at p < 0.05. Additional statistical details are provided in the Statistics Table S1.

#### Statistical analysis of human cohort data

Statistical analysis was performed in SPSS (version 28). To test differences between two groups Mann-Whitney or Chi-square tests were used. Spearman correlation analyses were carried out with two-tailed significance testing and computation of exact correlation coefficients (Spearman’s r).

## Supplemental information

Document S1. Figures S1–S9

Table S1. Excel file containing additional statistics information, related to Figures 1, 2, 3, 4, 5, 6, and S1–S9
