# Supplementary material for: Spatiotemporal sphingosine-1-phosphate receptor 3 expression within the cerebral vasculature after ischemic stroke
Source: iScience. 2024 May 20;27(6):110031. doi: 10.1016/j.isci.2024.110031 (PMC11167442; doi:10.1016/j.isci.2024.110031)
Supplement: Document S1. Figures S1–S9 [file mmc1.pdf]

iScience, Volume *27*

Supplemental information

Spatiotemporal sphingosine-1-phosphate receptor 3

expression within the cerebral

vasculature after ischemic stroke

Hana Matuskova, Lisa T. Porschen, Frank Matthes, Arne G. Lindgren, Gabor C. Petzold, and Anja Meissner

Supplemental Figures

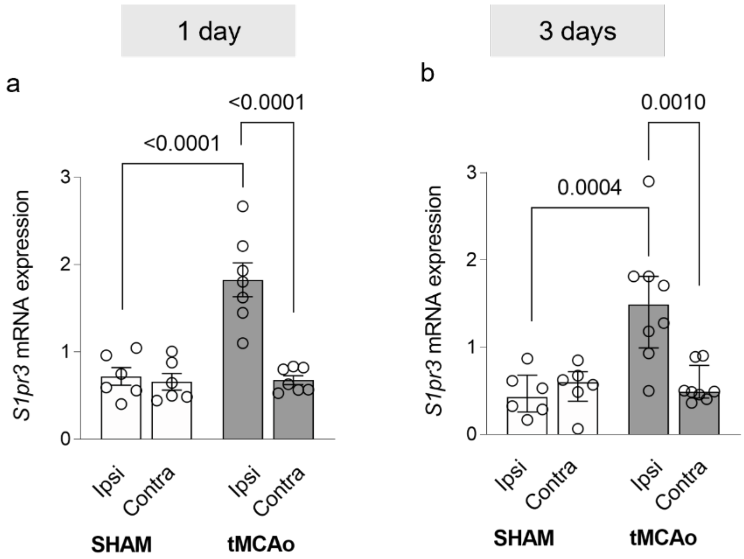

Figure S1:

***S1pr3* mRNA expression is augmented in mice post-stroke, related to Figure 1.** Transient middle cerebral artery occlusion (tMCAo) increases ipsilateral (ipsi) mRNA expression of sphingosine-1-phosphate receptor 3 (*S1pr3*) **(a)** 1-day (sham: n = 6; tMCAo: n = 7) and **(b)** 3-days (sham: n = 6; tMCAo: n = 8) post-stroke compared to contralateral (contra) and sham-operated controls. *Panel A* is presented as mean ± SEM and is compared with a Student's t-test. *Panel B* is presented as median ± interquartile range and is compared with 2-Way ANOVA and Sidak post-hoc testing. Exact p-values are given.

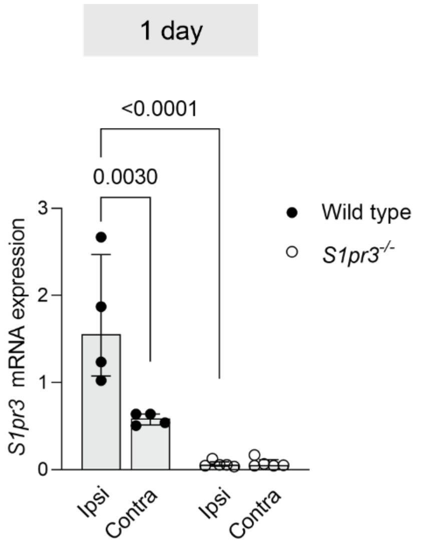

Figure S2:

***S1pr3* mRNA expression is augmented post-stroke in wild-type but not *S1pr3* knockout mice, related to Figure 1.** Ipsilateral (ipsi) sphingosine-1-phosphate receptor 3 (*S1pr3*) elevation in response to transient middle cerebral artery occlusion (tMCAo) is absent in *S1pr3* knockout mice (n = 5) compared to wild-type mice (n = 4). Data is presented as median ± interquartile range and is compared with a 2-Way ANOVA and Sidak post-hoc testing. Exact p-values are given.

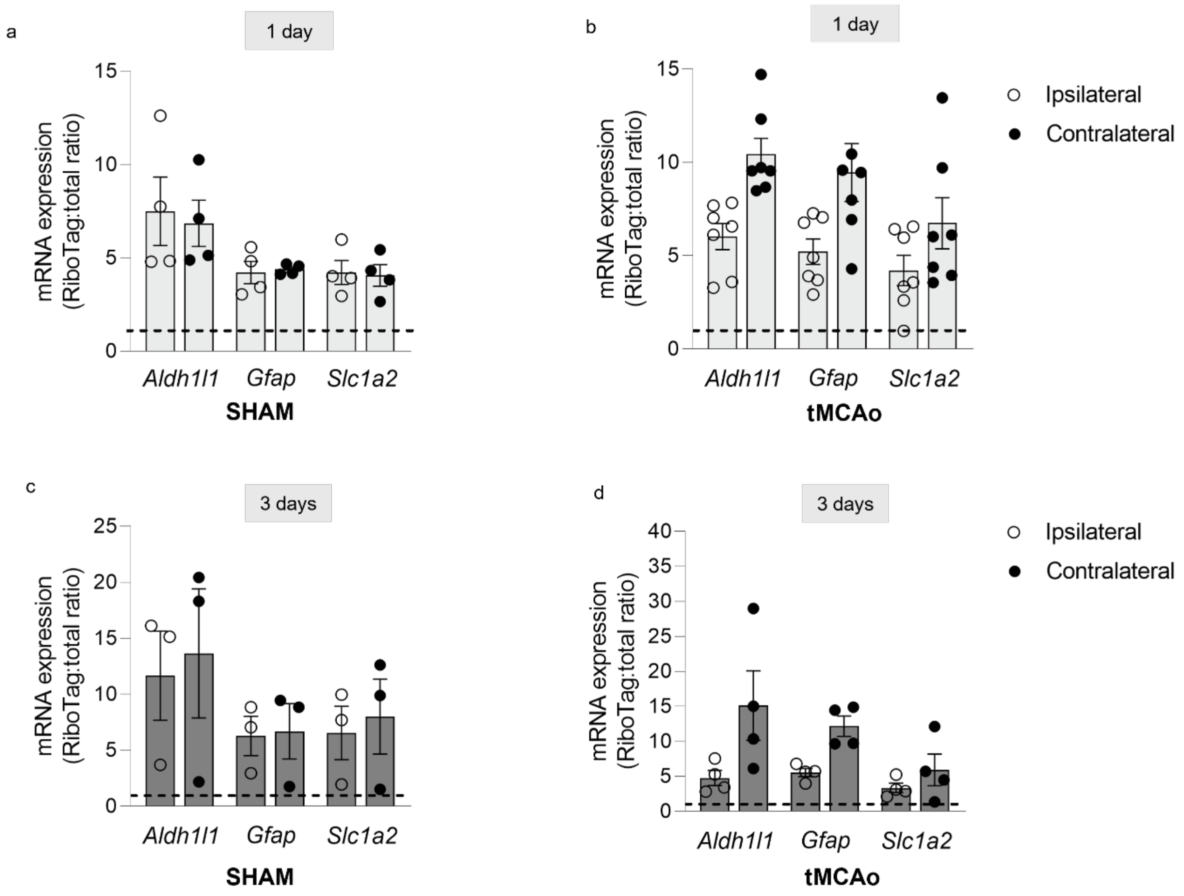

Figure S3:

Expression of astrocytic markers is enriched in immunoprecipitated mRNA from *Cnx43*$^{\text{Cre-ER(T)}}$/RiboTag mice, related to Figure 3. The ratio of immunoprecipitated to total mRNA expression of astrocytic markers *Aldh1l1*, *Gfap* and *Slc1a2*, is increased in sham and tMCAo at 1-day **(a);** (n = 4), **(b)**; (n = 7) and 3-days **(c);** (n = 3), **(d);** (n = 4) time points. *Panels A - D* are presented as mean ± SEM and are not subjected to statistical testing.

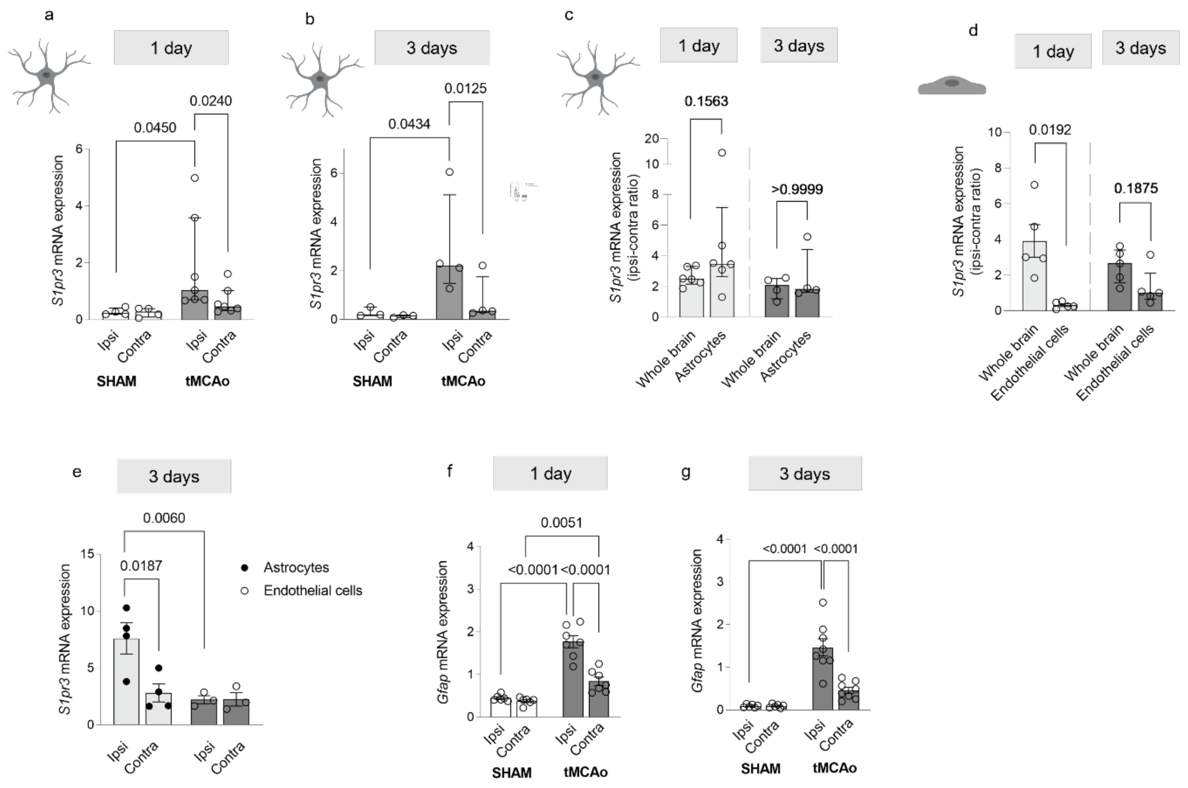

Figure S4:

***S1pr3* mRNA expression is augmented in mice post-stroke, related to Figure 3.** Astrocyte-specific sphingosine-1-phosphate receptor 3 (*S1pr3*) mRNA expression increases in response to transient middle cerebral artery occlusion (tMCAo) **(a)** 1-day (sham: n = 4; tMCAo: n = 7) and **(b)** 3-days (sham: n = 3; tMCAo: n = 4) post-stroke compared to control hemisphere (contra) and sham. **(c)** Ipsi-contra ratios of *S1pr3* expression is greater in astrocytes compared to whole brain tissue (1 day: n = 6; 3 days: n = 4). **(d)** Ipsi-contra ratios of *S1pr3* expression is smaller in endothelial cells compared to whole brain tissue (1 day: n = 5; 3 days: n = 5). **(e)** Ipsilateral *S1pr3* mRNA expression is upregulated in response to tMCAo in astrocytes (n = 4) but not in endothelial cells (n = 3). tMCAo increases ipsilateral (ipsi) mRNA expression of glial fibrillary acidic protein (*Gfap*) **(f)** 1-day (sham: n = 6; tMCAo: n = 7) and **(g)** 3-days (sham: n = 6; tMCAo: n = 8) post-stroke compared to sham-operated controls. *Panels A and B* are presented as median ± interquartile range and are compared with a 2-Way ANOVA and Sidak post-hoc testing. *Panels C and D (3-days)* are presented as median ± interquartile range and are compared with a Wilcoxon test. *Panel D (1-day) is presented as* mean ± SEM and is compared with Wilcoxon test. *Panels E, F, G* are presented as mean ± SEM and are compared with a 2-Way ANOVA and Sidak post-hoc testing. Exact p-values are given.

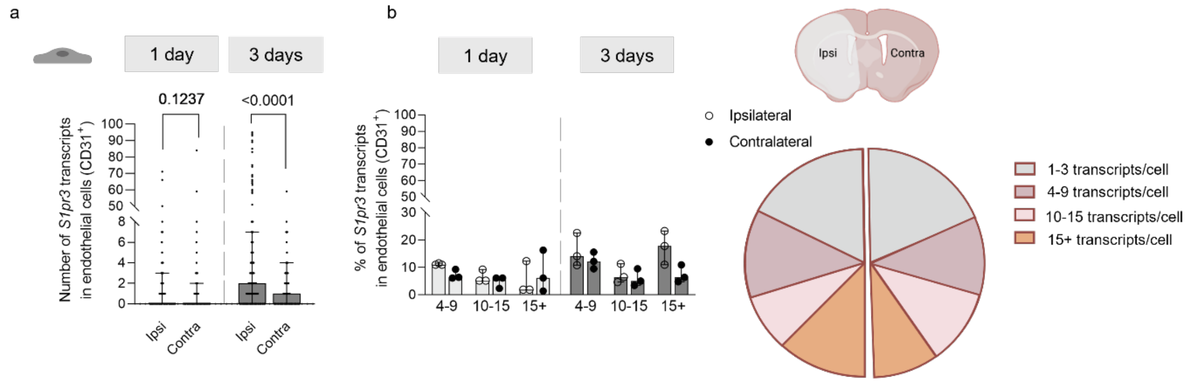

Figure S5:

Ipsilateral endothelial cell *S1pr3* transcript number is higher 3-days post-stroke, related to Figure 3. (a) Transient middle cerebral artery occlusion (tMCAo) does not affect ipsilateral (ipsi) transcript number of sphingosine-1-phosphate receptor 3 (*S1pr3*) per *CD31*$^{+}$ cell 1-day (n = 3) and increases the overall transcript number per *CD31*$^{+}$ cell 3-days post-stroke (n = 3) compared to contralateral (contra) hemisphere. **(b)** Categorization of *CD31*$^{+}$ cells according to *S1pr3* transcript number shows no differences between ipsilateral (ipsi) and contralateral (contra) hemispheres at 1-day or 3-days post-stroke. *Panel A* is presented as median ± interquartile range and is compared with a Mann-Whitney test. *Panel B* is presented as median ± interquartile range. Exact p-values are given.

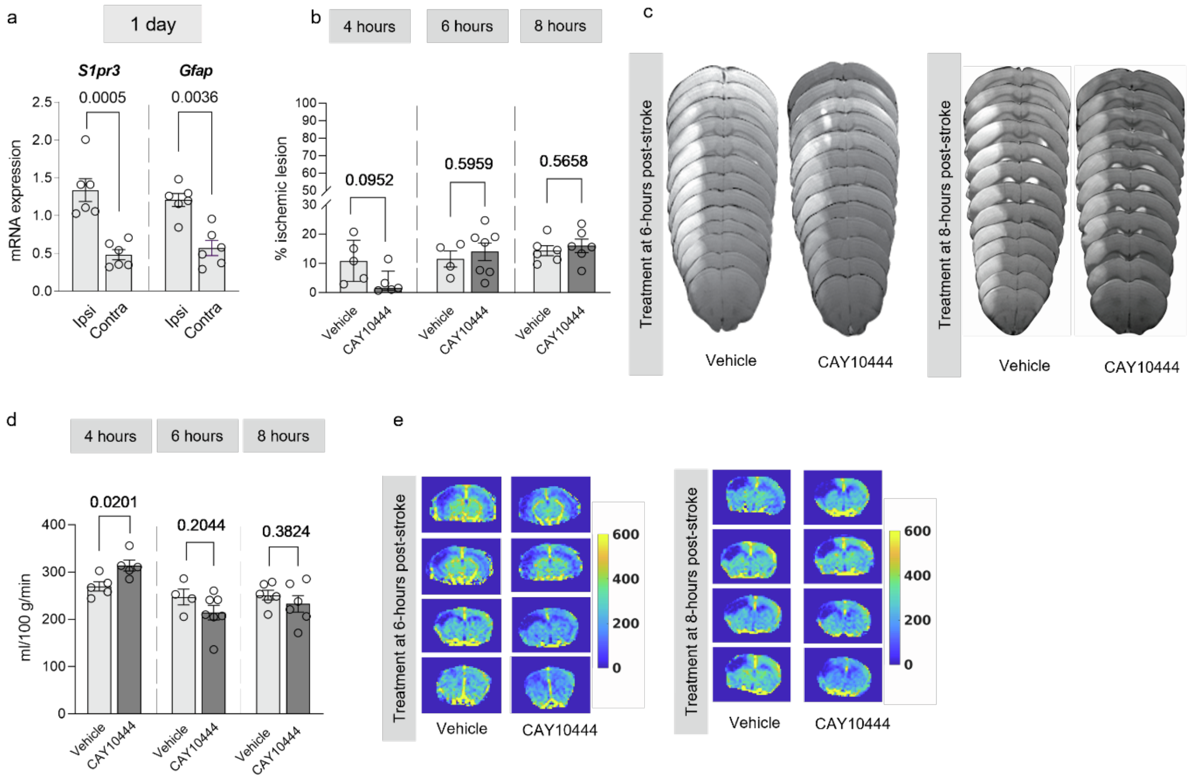

Figure S6:

Therapeutically antagonizing S1PR3 in mice improves stroke outcome dependent on time point of treatment initiation, related to Figure 4. (a) Permanent middle cerebral artery occlusion increases ipsilateral (ipsi) mRNA expression of sphingosine-1-phosphate receptor 3 (*S1pr3*) and glial fibrillary acidic protein (*Gfap*) 1-day (n= 6) post-stroke compared to the respective contralateral (contra) hemispheres. **(b)** Administration of the S1PR3 antagonist CAY10444 reduces overall infarct lesion at 1-day post-stroke only when given at 4-hours (4 hours: n = 5; 6 hours: Vehicle – n = 4, CAY10444 – n = 7; 8 hours: N = 6) post-stroke. **(c)** Representative T2 map images of infarct lesion for treatment initiated at 6- or 8-hours post-stroke. **(d)** Administration of the S1PR3 antagonist CAY10444 improves brain blood flow at 1-day post-stroke only when given at 4-hours (4 hours: n = 5; 6 hours: Vehicle – n = 4, CAY10444 – n = 7; 8 hours: n = 6) post-stroke. **(e)** Representative arterial spin labeling maps for treatments initiated at 6- or 8-hours post-stroke. *Panels A, B (6-, 8-hours) and D* are presented as mean ± SEM and are compared with a Student's t-test. *Panel B (4-hours)* is presented as median ± interquartile range and is compared with a Mann-Whitney test. Exact p-values are given.

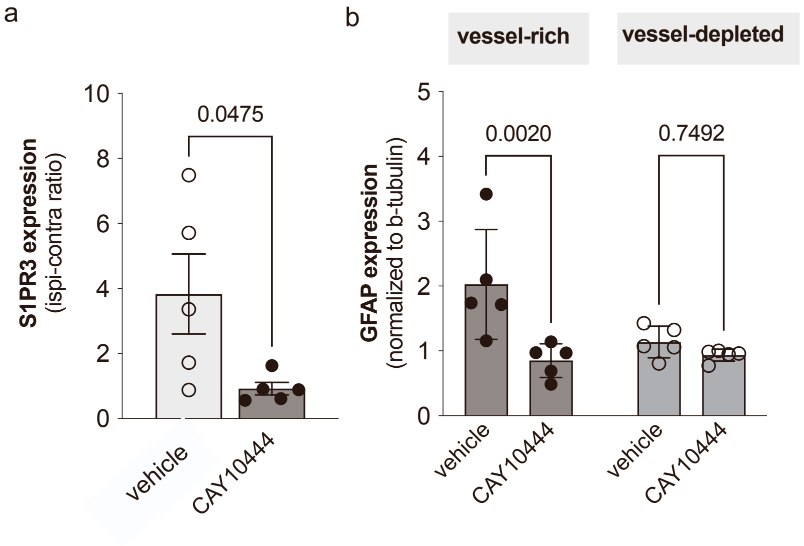

Figure S7:

S1PR3 antagonism mitigates stroke-induced vessel-associated S1PR3 upregulation and ipsilateral GFAP increases, related to Figure 4. (a) S1PR3 antagonism attenuates the increase of ipsilateral S1PR3 expression in the treated group (n = 5) compared to vehicle group (n = 5) in the vessel-rich fraction. **(b)** S1PR3 inhibitor downregulates ipsi:contra ratios of GFAP protein expression compared to the vehicle control in the vessel-rich fraction but has no effect in the vessel-depleted fraction. *Panels A and B are* presented as mean ± SEM and are compared with Student's t-test. Exact p-values are given.

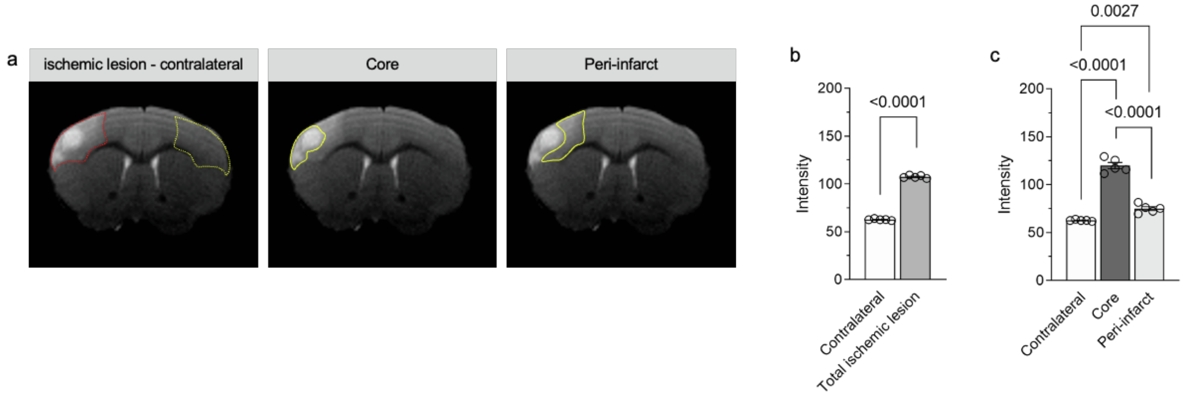

Figure S8:

Therapeutically antagonizing S1PR3 in mice reduces core area within the ischemic lesion dependent on time point of treatment initiation, related to Figure 4. Example of a T2 image chosen for core and peri-infract area definition **(a)**. The total ischemic lesion is determined as well-delineated area (in a, outlined in red) where **(b)** the increase of the T2 signal is significantly higher than the variation observed in the healthy contralateral tissue (n = 5). Core tissue was defined as an area with highest T2 signal within the ischemic lesion. In the representative image, the core region is manually drawn on the outline of the high intensity T2 image area (outlined in yellow). The peri-infarct area is considered as the area within the total ischemic lesion with significant lower T2 signal intensities compared to the core. **(c)** The so-defined peri-infarct region represented 70.91 ± 4.28 % of the core intensity. To adjust for inter-samples differences, we defined “peri-infarct area” as 80% of core intensity for analysis (n = 5). *Panels B* and *C* are presented as mean ± SEM and are compared with a Student’s t-test or 1-Way ANOVA, respectively. Exact p-values are presented.

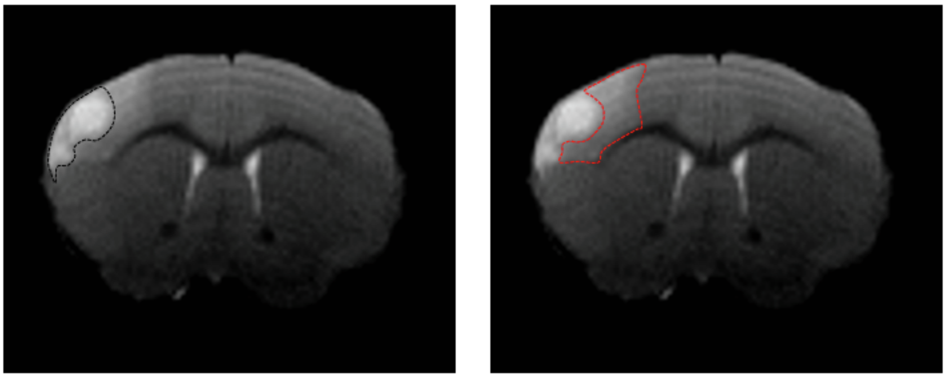

Figure S9:

Analysis of areas of infarct lesion with different level of damage, related to Figure 4, S8. To analyze areas with different water content (highlighted on the image below with black dash and red dash lines, respectively), first the outside area was cleared, and the resulting image was saved as *tiff* file. Using ImageJ, the threshold for brightness/contrast of all images was set to 128. All the visibly bright areas other than the infarct lesion were removed and data files with numbers of pixels for each value based on the corresponding histogram 0 – 255 /per each set of images were generated.
